# Publisher Correction: Improving the accuracy of medical diagnosis with causal machine learning

**DOI:** 10.1038/s41467-020-18310-1

**Published:** 2020-09-16

**Authors:** Jonathan G. Richens, Ciarán M. Lee, Saurabh Johri

**Affiliations:** 1 Babylon Health, 60 Sloane Ave, Chelsea, London, SW3 3DD UK; 2 University College London, Gower St, Bloomsbury, London, WC1E 6BT UK

**Keywords:** Predictive medicine, Diagnosis, Statistics

Correction to: *Nature Communications* 10.1038/s41467-020-17419-7, published online 11 August 2020.

The original version of the Source Data File associated with this Article was updated shortly after publication to redact sensitive intellectual property. During the update to the source data file, the Article incorrectly gave a publication date of 6 August 2020 in the HTML version; this should have been 11 August 2020. This has now been corrected in the HTML version of the Article. The PDF version of the Article was correct since the time of publication.

