# Author Correction: Improving the accuracy of medical diagnosis with causal machine learning

**DOI:** 10.1038/s41467-021-21494-9

**Published:** 2021-03-31

**Authors:** Jonathan G. Richens, Ciarán M. Lee, Saurabh Johri

**Affiliations:** 1 Babylon Health, 60 Sloane Ave, Chelsea, London, SW3 3DD UK; 2 University College London, Gower St, Bloomsbury, London, WC1E 6BT UK

**Keywords:** Predictive medicine, Diagnosis, Statistics

Correction to: *Nature Communications* 10.1038/s41467-020-17419-7, published online 11 August 2020.

The original version of this Article contained errors in the Data Availability statement, the Code Availability statement, the Competing Interests statement, Table 1 and Supplementary Table 2.

The Data availability statement read “The data that support the findings of this study are available on request from the corresponding author J.G.R. The data are not publicly available due to containing doctor and patient confidential information, as well as proprietary models. All data access requests will be reviewed and (if successful) granted by the Babylon Health Data Access Committee. Source data are provided with this paper.”

This has been corrected to “The data that support the findings of this study are available on https://github.com/babylonhealth/counterfactual-diagnosis. Any features of the vignettes not used to generate the methods or results of the study have been removed or de-identified prior to sharing. Accredited researchers may request access to the complete clinical dataset for the purpose of checking the validity of the clinical vignettes used in the study by contacting the corresponding author. Access will be vetted by the Babylon Health access committee and will take place within the Babylon health intranet and under a non-disclosure agreement.”

The Code availability statement read “The code used to generate results shown in this study is available from the author J.G.R. upon request.”

This has been corrected to “The code used to generate results shown in this study is available at https://github.com/babylonhealth/counterfactual-diagnosis.”

Additionally, reference 87 has been added to the reference list with a DOI for the public code associated with this Article: “87. Jonathan G. Richens, Ciaran M. Lee, Saurabh Johri, Improving the accuracy of medical diagnosis with causal machine learning, Zenodo, 10.5281/zenodo.4575649, 2021”

The Competing interests statement reported no competing interests for the authors; this has been corrected to “All the authors in the article are employees of Babylon Health.”

Also, one column was missing in Table 1, reporting data on “Extremely Rare” cases, and the “N” value for the Very Common vignettes was incorrectly repeated in the Draws row. The original version of Table 1 read:

**Table:** 

	All	VCommon	Common	Uncommon	Rare	VRare
*N*	1671	131	413	546	353	210
Mean position (A)	3.81	2.85	2.71	3.72	4.35	5.45
Mean position (C)	3.16	2.5	2.32	3.01	3.72	4.38
Wins (A)	31	2	7	9	9	4
Wins (C)	412	20	80	135	103	69
Draws	1228	131	326	402	241	137

This has been corrected to:

**Table:** 

	All	VCommon	Common	Uncommon	Rare	VRare	ERare
*N*	1671	131	413	546	353	210	18
Mean position (A)	3.81	2.85	2.71	3.72	4.35	5.45	4.22
Mean position (C)	3.16	2.5	2.32	3.01	3.72	4.38	3.56
Wins (A)	31	2	7	9	9	4	0
Wins (C)	412	20	80	135	103	69	5
Draws	1228	109	326	402	241	137	13

The third sentence of the legend of Table 1 previously read: “Results are stratified over the rareness of the disease (given the age and gender of the patient), where VCommon = Very common and VRare = very rare, and All is over all 1671 vignettes regardless of disease rarity”. This has now been corrected to: “Results are stratified over the rareness of the disease (given the age and gender of the patient), where VCommon = Very common, VRare = very rare and ERare = extremely rare, and All is over all 1671 vignettes regardless of disease rarity”. These have been corrected in both the PDF and HTML versions of the Article.

Supplementary Table 2 also reported statistics on “Extremely Rare” vignettes as “Almost Impossible” and included the same error of repeating *N* values in the Draws cell for Very Common vignettes. The original version of Supplementary Table 2 read:

**Table:** 

	All	Very common	Common	Uncommon	Rare	Very rare	Almost impossible
*N*	1671	131	413	546	353	210	18
Mean position (A)	3.81 ± 5.25	2.85 ± 4.27	2.71 ± 3.86	3.72 ± 5.05	4.35 ± 5.28	5.45 ± 6.52	4.22 ± 5.19
Mean position (C)	3.16 ± 4.40	2.5 ± 3.55	2.32 ± 3.25	3.01 ± 4.07	3.72 ± 4.74	4.38 ± 5.53	3.56 ± 3.96
Wins (A)	31	2	7	9	9	4	0
Wins (C)	412	20	80	135	103	69	5
Draws	1228	131	326	402	241	137	13

This has been corrected to:

**Table:** 

	All	Very common	Common	Uncommon	Rare	Very rare	Extremely rare
*N*	1671	131	413	546	353	210	18
Mean position (A)	3.81 ± 5.25	2.85 ± 4.27	2.71 ± 3.86	3.72 ± 5.05	4.35 ± 5.28	5.45 ± 6.52	4.22 ± 5.19
Mean position (C)	3.16 ± 4.40	2.5 ± 3.55	2.32 ± 3.25	3.01 ± 4.07	3.72 ± 4.74	4.38 ± 5.53	3.56 ± 3.96
Wins (A)	31	2	7	9	9	4	0
Wins (C)	412	20	80	135	103	69	5
Draws	1228	109	326	402	241	137	13

The HTML has been updated to include a corrected version of the Supplementary Information. The original HTML version of the paper incorrectly reported in the first paragraph of the Methods subsection “Noisy-OR twin diagnostic networks” that “Concretely, the value s of S is the Boolean OR function ∧ of its parents activation functions, s = ∧ if(di, ui), where the activation functions take the form f(di,ui)=di∧u¯i, ∨ denotes the Boolean AND function, di ∈ {0, 1} is the state of a given parent Di and ui ∈ {0, 1} is a latent noise variable (u¯i:=1−ui) with a probability of failure P(ui=1)=λDi,S.” The OR and the AND symbols, respectively ∨ and ∧, have been swapped. It now reads, “Concretely, the value s of S is the Boolean OR function ∨ of its parents activation functions, s = ∨ if(di, ui), where the activation functions take the form f(di,ui)=di∧u¯i, ∧ denotes the Boolean AND function, di ∈ {0, 1} is the state of a given parent Di and ui ∈ {0, 1} is a latent noise variable (u¯i:=1−ui) with a probability of failure P(ui=1)=λDi,S.” This has been corrected in the HTML version of the Article; the PDF version was correct from the time of publication.

## Supplementary information

Supplementary Materials

