# Supplementary material for: Improving the accuracy of medical diagnosis with causal machine learning
Source: Nat Commun. 2020 Aug 11;11:3923. doi: 10.1038/s41467-020-17419-7 (PMC7419549; doi:10.1038/s41467-020-17419-7)
Supplement: Supplementary file 1 — Supplementary Information [file 41467_2020_17419_MOESM1_ESM.pdf]

Supplementary Materials: Improving the accuracy of medical diagnosis with causal machine learning. Richens et. al.

The structure of these supplementary notes is as follows. In supplementary note 1 we detail our notation. In supplementary note 2 we outline the tools we use to derive our results – namely the frameworks of structural causal models (SCMs), introduce noisy-or Bayesian networks, and derive their SCM representation. In supplementary note 3 we outline the framework of twin-networks [1], and derive a simplified class of twin networks that we will use for computing our counterfactual diagnostic measures ('twin diagnostic networks'). In supplementary notes 4 and 6 we introduce and derive expressions for our counterfactual diagnostic measures—the expected sufficiency and the expected disablement—for the family of noisy-or diagnostic networks introduced in supplementary notes 2 and 3. In supplementary notes 5 and 7 we prove that these two measures satisfy our desiderata. In supplementary note 8 we discuss the expected disablement and sufficiency in relation to other counterfactual measures. In supplementary note 9 we look at simple diagnostic scenarios where the posterior leads to spurious diagnoses, and show how the expected disablement and sufficiency overcome this and why the expected disablement and sufficiency achieve a similar accuracy on our test set. In supplementary note 10 we provide an example of our clinical vignettes. Finally, in the supplementary tables we list our experimental results.

Supplementary Notes

Supplementary note 1: notation

Variables: For the disease models we consider, all variables X are Bernoulli, $X \in \{0, 1\}$. Where appropriate we refer to $X = 0$ as the variable X being 'off', and $X = 1$ as the variable X being 'on'. We denote single variables as capital Roman letters, and sets of variables as calligraphic, e.g. $\mathcal{X} = \{X_1, X_2, \ldots, X_n\}$. The union of two sets of variables $\mathcal{X}$ and $\mathcal{Y}$ is denoted $\mathcal{X} \cup \mathcal{Y}$, the intersection is denoted $\mathcal{X} \cap \mathcal{Y}$, and the relative compliment of $\mathcal{X}$ w.r.t $\mathcal{Y}$ as $\mathcal{X} \setminus \mathcal{Y}$. The instantiation of a single variable is indicated by a lower case letter, $X = x$, and for a set of variables $\mathcal{X} = \underline{x}$ denotes some arbitrary instantiation of all variables belonging to $\mathcal{X}$, e.g. $X_1 = x_1, X_2 = x_2, \ldots, X_n = x_n$. The probability of $\mathcal{X} = \underline{x}$ is denoted $P(\mathcal{X} = \underline{x})$, and sometimes for simplicity is denoted as $P(\underline{x})$.

For a given variable X and a directed acyclic graph (DAG) G, we denote the set of parents of X as $\mathsf{Pa}(X)$, the set of children of X as $\mathsf{Ch}(X)$, all ancestors of X as $\mathsf{Anc}(X)$, and all descendants of X as $\mathsf{Dec}(X)$. If we perform a graph cut operation on G, removing a directed edge from Y to X, we denote the variable X in the new DAG generated by this cut as $X^{\setminus Y}$.

Functions: Bernoulli variables are represented interchangeably as Boolean variables, with $1 \leftrightarrow$ 'True' and $0 \leftrightarrow$ 'False'. For a given instantiation of a Bernoulli/Boolean variable $X = x$, we denote the negation of x as $\bar{x}$ – for example if $x = 1(0)$, $\bar{x} = 0(1)$. We denote the Boolean AND function as $\wedge$, and the Boolean OR function as $\vee$.

Supplementary note 2: structural causal models

First we define structural causal models (SCMs), sometimes also called structural equation models or functional causal models. These are widely applied and studied probabilistic models, and their relation to other approaches such as Bayesian networks are well understood [2, 3]. The key characteristic of SCMs is that they represent variables as functions of their direct causes, along with an exogenous 'noise' variable that is responsible for their randomness.

Definition 1 (Structural Causal Model). *A causal model specifies:*

1. *a set of latent, or noise, variables* $\mathcal{U} = \{u_1, \ldots, u_n\}$*, distributed according to* $P(\mathcal{U})$*.*
2. *a set of observed variables* $\mathcal{V} = \{v_1, \ldots, v_n\}$*,*
3. *a directed acyclic graph* G*, called the causal structure of the model, whose nodes are the variables* $\mathcal{U} \cup \mathcal{V}$*,*
4. *a collection of functions* $F = \{f_1, \ldots, f_n\}$*, where* f_i *is a mapping from* $\mathcal{U} \cup \mathcal{V}/v_i$ *to* v_i*. The collection* F *forms a mapping from* $\mathcal{U}$ *to* $\mathcal{V}$*. This is symbolically represented as*

$$v_i = f_i(\mathsf{Pa}(v_i), u_i), \text{ for } i = 1, \ldots, n,$$

where pa_i *denotes the parent nodes of the ith observed variable in* G*.*

Note that the causal structure and generative functions are typically provided by expert opinion, though in some instances the causal structure can be learned from data [4, 5]. As the collection of functions F forms a mapping from noise variables $\mathcal{U}$ to observed variables $\mathcal{V}$, the distribution over noise variables induces a distribution over observed variables, given by

$$P(v_i) := \sum_{u \mid v_i = f_i(\mathsf{Pa}(v_i), u)} P(u), \text{ for } i = 1, \ldots, n. \tag{1}$$

We can hence assign uncertainty over observed variables despite the the underlying dynamics being deterministic.

In order to formally define a counterfactual query, we must first define the interventional primitive known as the *do*-operator [3]. Consider a SCM with functions F. The effect of intervention $do(X = x)$ in this model corresponds to creating a new SCM with functions $F_{X=x}$, formed by deleting from F all functions f_i corresponding to members of the set X and replacing them with the set of constant functions $X = x$. That is, the *do*-operator forces variables to take certain values, regardless of the original causal mechanism. This represents the operation whereby an agent intervenes on a variable, fixing it to take a certain value. Probabilities involving the *do*-operator, such as $P(Y = y|do(X = x))$, correspond to evaluating ordinary probabilities in the SCM with functions $F_{X=x}$, in this case $P(Y = y)$. Where appropriate, we use the more compact notation of Y_x to denote the variable Y following the intervention $\text{do}(X = x)$.

Next we define noisy-OR models, a specific class of SCMs for Bernoulli variables that are widely employed as diagnostic models [6–15]. The noisy-OR assumption states that a variable Y is the Boolean OR of its parents $X_1, X_2, \ldots, X_n$, where the inclusion or exclusion of each causal parent in the OR function is decided by an independent probability or 'noise' term. The standard approach to defining noisy-OR is to present the conditional independence constraints generated by the noisy-OR assumption [16],

$$P(Y = 0 \mid X_1, \ldots, X_n) = \prod_{i=1}^{n} P(Y = 0 \mid \text{only}(X_i = 1)) \tag{2}$$

where $P(Y = 0 \mid \text{only}(X_i = 1))$ is the probability that $Y = 0$ conditioned on all of its (endogenous) parents being 'off' ($X_j = 0$) except for X_i alone. We denote $P(Y = 0 \mid \text{only}(X_i = 1)) = \lambda_{X_i,Y}$ by convention.

The utility of this assumption is that it reduces the number of parameters needed to specify a noisy-OR network to $\mathcal{O}(N)$ where N is the number of directed edges in the network. All that is needed to specify a noisy-OR network are the single variable marginals $P(X_i = 1)$ and, for each directed edge $X_i \rightarrow Y_j$, a single λ_{X_i,Y_j}. For this reason, noisy-OR has been a standard assumption in Bayesian diagnostic networks, which are typically large and densely connected and so could not be efficiently learned and stored without additional assumptions on the conditional probabilities. We now define the noisy-OR assumption for SCMs.

Definition 2 (noisy-OR SCM)**.** *A noisy-OR network is an SCM of Bernoulli variables, where for any variable Y with parents $\mathsf{Pa}(Y) = \{X_1, \ldots, X_N\}$ the following conditions hold*

1. *Y is the Boolean OR of its parents, where for each parent X_i there is a Bernoulli variable U_i whose state determines if we include that parent in the OR function or not*

$$y = \bigvee_{i=1}^{N} (x_i \wedge \bar{u}_i) \tag{3}$$

i.e. $Y = 1$ if any parent is on, $x_i = 1$, and is not ignored, $u_i = 0$ ($\bar{u}_i = 1$ where 'bar' denotes the negation of u_i).

2. *The exogenous latent encodes the likelihood of ignoring the state of each parent in (1), $P(u_Y) = P(u_1, u_2, \ldots, u_N)$. The probability of ignoring the state of a given parent variable is independent of whether you have or have not ignored any of the other parents,*

$$P(u_1, u_2, \ldots, u_N) = \prod_{i=1}^{N} P(u_i)$$

3. *For every node Y there is a parent 'leak node' L_Y that is singly connected to Y and is always 'on', with a probability of ignoring given by λ_{L_Y}*

The leak node (assumption 3) represents the probability that $Y = 1$, even if $X_i = 0 \ \forall \ X_i \in \mathsf{Pa}(Y)$. This allows $Y = 1$ to be caused by an exogenous factor (outside of our model). For example, the leak nodes allow us to model the situation that a disease spontaneously occurs, even if all risk factors that we model are absent, or

that a symptom occurs but none of the diseases that we model have caused it. It is conventional to treat the leak node associated with a variable Y as a parent node L_Y with $P(L_Y = 1)$. Every variable in the noisy-OR SCM has a single, independent leak node parent.

Given Definition 2, why is the noisy-or assuption justified for modelling diseases? First, consider the assumption (1), that the generative function is a Boolean OR of the individual parent 'activation functions' $x_i \cap \bar{u}_i$. This is equivalent to assuming that the activations from diseases or risk-factors to their children never 'destructively interfere'. That is, if D_i is activating symptom S, and so is D_j, then this joint activation never cancels out to yield $S = F$. As a consequence, all that is required for a symptom to be present is that at least one disease to be causing it, and likewise for diseases being caused by risk factors. This property of noisy-OR, whereby an individual cause is also a sufficient cause, is a natural assumption for diseases modelling – where diseases are (typically by definition) sufficient causes of their symptoms, and risk factors are defined such that they are sufficient causes of diseases. For example, if preconditions $R_1 = 1$ and $R_2 = 1$ are needed to cause $D = 1$, then we can represent this as a single risk factor $R = R_1 \wedge R_2$. Assumption 2 states that a given disease (risk factor) has a fixed likelihood of activating a symptom (disease), independent of the presence or absence of any other disease (risk factor). In the noisy-or model, the likelihood that we ignore the state of a parent X_i of variable Y_i is given by

$$P(u_i = 1) = \frac{P(Y_i = 0|\ \mathrm{do}(X_i = 1))}{P(Y_i = 0|\ \mathrm{do}(X_i = 0))} \tag{4}$$

and so is directly associated with a (causal) relative risk. In the case that child Y has two parents, X_1 and X_2, noisy-OR assumes that this joint relative risk factorises as

$$\begin{aligned} P(u_1 = 1, u_2 = 1) &= \frac{P(Y = 0|\ \mathrm{do}(X_1 = 1, X_2 = 1))}{P(Y = 0|\ \mathrm{do}(X_1 = 0, X_2 = 0))} = \frac{P(Y = 0|\ \mathrm{do}(X_1 = 1))}{P(Y = 0|\ \mathrm{do}(X_1 = 0))} \times \frac{P(Y = 0|\ \mathrm{do}(X_2 = 1))}{P(Y = 0|\ \mathrm{do}(X_2 = 0))} && (5) \\ &= P(u_1 = 1)P(u_2 = 1) && (6) \end{aligned}$$

Whilst it is likely that interactions between causal parents will mean that these relative risks are not always multiplicative, it is assumed to be a good approximation. For example, we assume that the likelihood that a disease fails to activate a symptoms is independent of whether or not any other disease similarly fails to activate that symptom.

As noisy-OR models are typically presented as Bayesian networks, the above definition of noisy-OR is non-standard. We now show that the SCM definition yields the Bayesian network definition, (2).

Theorem 1 (noisy-OR CPT)**.** *The conditional probability distribution of a child Y given its parents $\{X_1, \ldots, X_N\}$ and obeying Definition 2 is given by*

$$P(Y = 0 \,|\, X_1 = x_1, \ldots, X_n = x_N) = \prod_{i=1}^{N} \lambda_{X_i,Y}^{x_i} \tag{7}$$

where

$$\lambda_{X_i,Y} = P(Y = 0|\ \textit{only}\ (X_i = 1)) \tag{8}$$

Proof. For $Y = 0$, the negation of y, denoted $\bar{y}$, is given by

$$\bar{y} = \neg \left(\bigvee_{i=1}^{N} (x_i \wedge \bar{u}_i) \right) = \bigwedge_{i=1}^{N} (\bar{x}_i \vee u_i) \tag{9}$$

The CPT is calculated from the structural equations by marginalizing over the latents, i.e. we sum over all latent states that yield $Y = 0$. Equivalently, we can marginalize over all exogenous latent states multiplied by the above Boolean function, which is 1 if the condition $Y = 0$ is met, and 0 otherwise.

$$
\begin{aligned}
P(Y=0\,|\,X_1=x_1,\ldots,X_n=x_n) &= \sum_{u_1}\cdots\sum_{u_N}\bigwedge_{i=1}^{N}(\bar{x}_i\vee u_i)\,P(u_Y)\\
&= \sum_{u_1}\cdots\sum_{u_N}\prod_{X_i}(\bar{x}_i\vee u_i)\prod_{U_i}P(u_i)\\
&= \prod_{X_i}\sum_{U_i=u_i}P(u_i)\,(\bar{x}_i\vee u_i)\\
&= \prod_{X_i}\left[P(u_i=1)+P(u_i=0)\bar{x}_i\right]\\
&= \prod_{X_i}\left[\lambda_{X_i,Y}+(1-\lambda_{X_i,Y})\bar{x}_i\right]\\
&= \prod_{X_i}\lambda_{X_i,Y}^{x_i} \qquad (10)
\end{aligned}
$$

This is identical to the noisy-OR CPT (2) □

where we denote $\lambda_{X_i,Y} = P(u_i)$. The leak node is included as a parent X_L where $P(X_L = 1) = 1$, and a (typically large) probability of being ignored λ_L. This node represents the likelihood that Y will be activated by some causal influence outside of the model, and is included to ensure that $P(Y = 1|\wedge_{i=1}^{n}(X_i = 0)) \neq 0$. As the leak node is always on, its notation can be suppressed and it is standard notation to write the CPT as

$$P(Y=0\,|\,X_1=x_1,\ldots,X_n=x_n) = \lambda_L\prod_{X_i}\lambda_{X_i,Y}^{x_i} \qquad (11)$$

Supplementary note 3: Twin diagnostic networks

In this supplementary note we derive the structure of diagnostic twin networks. First we provide a brief overview to the twin-networks approach to counterfactual inference. See [1] and [17] for more details on this formalism. First, recalling the definition of the do operator from the previous section, we define counterfactuals as follows.

Definition 3 (Counterfactual). *Let X and Y be two subsets of variables in V. The counterfactual sentence Y would be y (in situation U), had X been x, is the solution $Y = y$ of the set of equations F_x, succinctly denoted $Y_x(U) = y$.*

As with observed variables in Definition 1, the latent distribution $P(U)$ allows one to define the probabilities of counterfactual statements in the same manner they are defined for standard probabilities (1).

$$P(Y_x = y) = \sum_{u|Y_x(u)=y} P(u). \qquad (12)$$

Reference [3] provides an algorithmic procedure for computing arbitrary counterfactual probabilities for a given SCM. First, the distribution over latents is updated to account for the observed evidence. Second, the *do*-operator is applied, representing the counterfactual intervention. Third, the new causal model created by the application of the *do*-operator in the previous step is combined with the updated latent distribution to compute the counterfactual query. In general, denote $\mathcal{E}$ as the set of factual evidence. The above can be summarised as,

1. (abduction). The distribution of the exogenous latent variables $P(u)$ is updated to obtain $P(u\,|\,\mathcal{E})$
2. (action). Apply the do-operation to the variables in set X, replacing the equations $X_i = f_i(\mathrm{Pa}(x_i), u_i)$ with $X_i = x_i \;\forall\; X_i \in X$.
3. (prediction). Use the modified model to compute the probability of $Y = y$.

The issue with applying this approach to our large diagnostic models is that the first step, updating the exogenous latents, is in general intractable for models with large tree-width. The twin-networks formalism, introduced in [1], is a method which reduces and amortises the cost of this procedure. Rather than explicitly updating the exogenous latents, performing an intervention, and performing belief propagation on the resulting SCM, twin networks allow us to calculate the counterfactual by performing belief propagation on a single 'twin' SCM – without requiring the expensive abduction step. The twin network is constructed as a composite of two copies of the original SCM where copied variables share their corresponding latents [1]. We refer to pairs of copied variables as 'dual variables'. Nodes on this twin network can then be merged following simple

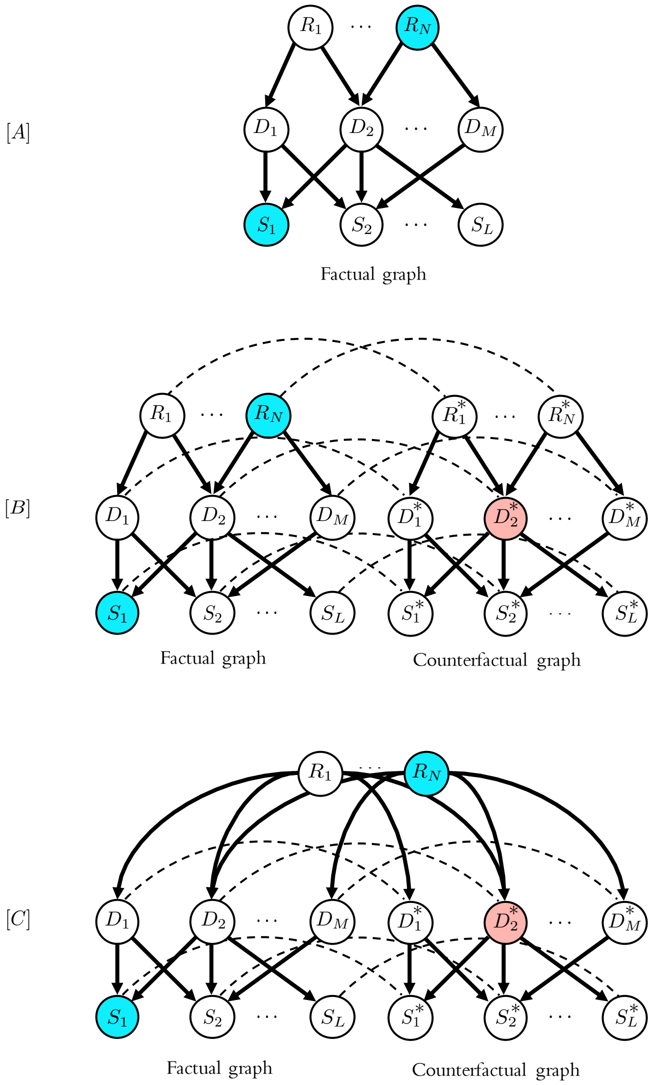

Supplementary Figure 1: Construction of twin network

rules outlined in [17], further reducing the complexity of computing the counterfactual query. We now outline the process of constructing the twin diagnostic network in the case of the two counterfactual queries we are interested in – those with single counterfactual interventions, and those where all counterfactual variables bar one are intervened on.

We assume the DAG structure of our diagnostic model is a three layer network [A]. The top layer nodes represent risk factors, the second layer represent diseases, and the third layer symptoms. We assume no directed edges between nodes belonging to the same layer. To construct the twin network, first the SCM in [A] is copied. In [B] the network on the left will encode the factual evidence in our counterfactual query, and we refer to this as the factual graph. The network on the right in [B] will encode our counterfactual interventions and observations, and we refer to this as the counterfactual graph. We use an asterisk X^* to denote the counterfactual dual variable of X.

As detailed in [1], the twin network is constructed such that each node on the factual graph shares its exogenous latent with its dual node, so $u^*_{X_i} = u_{X_i}$. These shared exogenous latents are shown as dashed lines in figures [B-E]. First, we consider the case where we perform a counterfactual intervention on a single disease. As shown in [B], we select a disease node in the counterfactual graph to perform our intervention on (in this instance D^*_2). In Figure [C], blue circles represent observations and red circles represent interventions. The do-operation severs any directed edges going into D^* and fixes $D^* = 0$, as shown in [D] below.

Once the counterfactual intervention has been applied, it is possible to greatly simplify the twin network graph structure via node merging [17]. In SCM's a variable takes a fixed deterministic value given an instantation of all of its parents and its exogenous latent. Hence, if two nodes have identical exogenous latents and parents, they are copies and can be merged into a single node. By convention, when we merge these identical dual nodes we map $X^* \mapsto X$ (dropping the asterisk). Dual nodes which share no ancestors that have been intervened upon can therefore be merged. As we do not perform interventions on the risk factor nodes, all (R_i, R^*_i) are merged

(note that for the sake of clarity we do not depict the exogenous latents for risk factors).

[D]

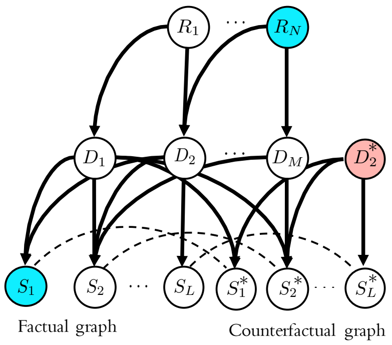

[E]

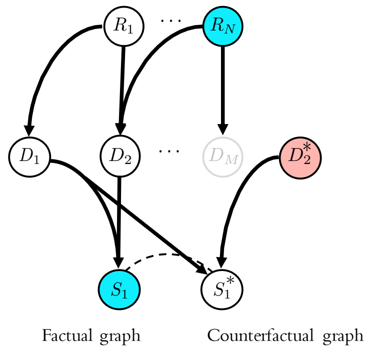

Supplementary Figure 2: Simplification of twin network through node merging

Next, we merge all dual factual/counterfactual disease nodes that are not intervened on, as their latents and parents are identical (shown in [D]). Finally, any symptoms that are not children of the disease we have intervened on (D_2) can be merged, as all of their parent variables are identical. The resulting twin network is shown in [E]. Note that we have also removed any superfluous symptom nodes that are unevidenced, as they are irrelevant for the query.

In the case that we intervene on all of the counterfactual diseases except one, following the node merging rule outlined above, we arrive at a model with a single disease that is a parent of both factual and counterfactual symptoms, as shown in Figure [F].

[F]

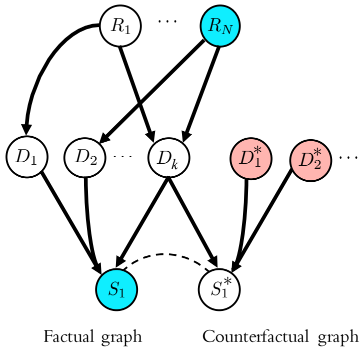

Supplementary Figure 3: Final twin network for expected sufficiency

We refer to the SCMs shown in figures [E] and [F] as 'twin diagnostic networks'. The counterfactual queries we are interested in can be determined by applying standard inference techniques such as importance sampling to these models [18].

Supplementary note 4: expected sufficiency

In this supplementary note we derive a simple closed form expression for our proposed diagnostic measure, the *expected sufficiency*, which corresponds to the case where we perform counterfactual interventions on all diseases bar one (D_k, model shown in Figure [F]). We derive our expressions for three layer noisy-OR SCM's.

Before proceeding, we motivate our choice of counterfactual query for the task of diagnosis.

An observation will often have multiple possible causes, which constitute competing explanations. For example, the observation of a symptom $S = 1$ can in principle be explained by any of its parent diseases. In the case that a symptom has multiple associated causes (diseases), rarely is a single disease *necessary* to explain a given symptom, unless the symptom is uniquely generated by the disease. Equivalently, the symptoms associated with a disease tend to be present in patient's suffering from this diseases, without *requiring* a secondary disease to be present. This can be summarised by the following assumption – *any single disease is a sufficient cause of any of its associated symptoms.* Under this assumption, determining the likelihood that a diseases is causing a symptom reduces to simple deduction – removing all other possible causes and seeing if the symptom remains. We call this the assumption of causal sufficiency and note that it is a standard assumption in most of medicine, and is often taken as part of the definition of the symptoms of a disease.

The question of how we can define and quantify causal explanations in general models is an area of active research [19–22] and the approach we propose here cannot be applied to all conceivable SCMs, as counterfactual inferences are valid only up to a set of modelling assumptions [23]. For example, if you had a symptom that can be present *only if* two parents diseases D_1 and D_2 are both present, then neither of these parents in isolation is a sufficient cause (individually, $D_1 = 1$ and $D_2 = 1$ are necessary but not sufficient to cause $S = 1$). This case would violate the assumption of causal sufficiency. In supplementary note 6 we present a different counterfactual query that does not require causal sufficiency, and captures causality in this case by reasoning about necessary treatments.

The assumption of causal sufficiency is obey by noisy-Or models, as in these models all diseases are *individually sufficient* to generate any symptom. This is ensured by the OR function, which states that a symptom S is the Boolean OR of its parents individual activation functions, $s = \bigvee_{i=1}^{N}[d_i \wedge \bar{u}_{D_i,S}]$ where the activation function from parent D_i is $f_i = d_i \wedge \bar{u}_{D_i,S}$. Thus, any single activation is sufficient to explain $S = 1$ and we can quantify the expected sufficiency of a diseases individually. An example of a model that would violate this property is a noisy-AND model, where $s = \bigwedge_{i=1}^{N}[d_i \wedge \bar{u}_{D_i,S}]$ - e.g. all parent diseases must be present in order for the symptom to be present.

Given these properties of noisy-OR models (as disease models in general), we propose our measure for quantifying how well a disease explains the patient's symptoms – the *expected sufficiency.* For a given disease, this measures the number of symptoms that we would expect to remain if we intervened to nullify all other possible causes of symptoms. This counterfactual intervention is represented by the causal model shown in figure [F] in supplementary note A.2.

Definition 2 *The expected sufficiency of disease D_k determines the number of positively evidenced symptoms we would expect to persist if we intervene to switch off all other possible causes of the symptoms,*

$$\mathbb{E}_{\text{suff}}(D_k, \mathcal{E}) := \sum_{\mathcal{S}'} \left|\mathcal{S}'_+\right| P(\mathcal{S}'|\mathcal{E}, \text{do}(\mathsf{Pa}(\mathcal{S}_+) \setminus D_k = 0)) \tag{13}$$

where the expectation is calculated over all possible counterfactual symptom evidence states $\mathcal{S}'$ and $\mathcal{S}'_+$ denotes the positively evidenced symptoms in the counterfactual symptom evidence state. $\mathsf{Pa}(\mathcal{S}'_+ \setminus D_k)$ denotes the set of all parents of the set of counterfactual positively evidenced symptoms $\mathcal{S}'_+$ excluding D_k, and $do(\mathsf{Pa}(\mathcal{S}_+) \setminus D_k = 0)$ denotes the counterfactual intervention setting $\mathsf{Pa}(\mathcal{S}'_+ \setminus D_k) \to 0$. $\mathcal{E}$ denotes the set of all factual evidence.

To evaluate the expected sufficiency we must first determine the dual symptom CPTs in the corresponding twin network (figure [F]).

Lemma 1. *For a given symptom S and its counterfactual dual S^*, with parent diseases $\mathcal{D}$ and under the counterfactual interventions $do(\mathcal{D} \setminus D^*_k = 0)$ and $do(\mathcal{U}^*_L = 0)$, the joint conditional distribution is given by*

$$P(s, s^* | \wedge_{i=1}^{N} d_i, do(\wedge_{i\neq k} D^*_i = 0), do(u^*_L = 0)) = \begin{cases} P(s = 0 | \wedge_{i=1}^{N} d_i), & s = s^* = 0 \\ 0, & s = 0, s^* = 1 \\ \lambda_{D_k,s}^{d_k} P(s^{\setminus k} = 1 | \wedge_{i\neq k} d_i, D_k = 1), & s = 1, s^* = 0 \\ (1 - \lambda_{D_k,S})\delta(d_k - 1), & s = 1, s^* = 1 \end{cases}$$

where $\delta(d_k - 1) = 1$ if $D_k = 1$ else 0, and $\underline{d}$ is an instantiation of all $D_i \in \mathsf{Pa}(S)$, $\wedge_{i\neq k} D^*_i$ is the set of all counterfactual disease nodes excluding D_k, $\wedge_{i\neq k} d_i$ is the given instantiation on all disease nodes exlcuding D_k, and u^*_L denotes the leak node for the counterfactual symptom. $s^{\setminus k}$ denotes the state of the factual symptom node S under the graph surgery removing any direct edge from D_k to S.

Proof. The CPT for the dual symptom nodes S, S^* is given by

$$\begin{aligned} &P(s, s^* | \underline{d}, \text{do}(\wedge_{i\neq k} D^*_i = 0), \text{do}(u^*_L = 0)) = \\ &\sum_{u_{D_1,S}} P(u_{D_1,S}) \cdots \sum_{u_{D_N,S}} P(u_{D_N,S}) \sum_{u_L} P(u_L) P(s | d_k, \wedge_{i\neq k} d_i, u_L) P(s^* | d_k, \text{do}(\wedge_{i\neq k} D^*_i = 0), \text{do}(u^*_L = 0)) \end{aligned} \tag{14}$$

Where we have use the fact that the latent variables and the disease variables together form a Markov blanket for S, S^*, and we have used the conditional independence structure of the twin network, shown in Figure [F], which implies that S and S^* only share a single variable, D_k, in their Markov blankets. With the full Markov blanket specified, including the exogenous latents, the CPTs in (14) are deterministic functions, each taking the value 1 if their conditional constraints are satisfied. Note that the product of these two functions is equivalent to a function that is 1 if both sets of conditional constraints are satisfied and zero otherwise, and marginalizing over all latent variable states multiplied by this function is equivalent to the definition of the CPT for SCMs given in equation (1), where the CPT is determined by a conditional sum over the exogenous latent variables. Given the definition of the noisy-OR SCM in (3), these functions take the form

$$P(s|d_k, \wedge_{i\neq k} d_i, u_L) = \begin{cases} \bar{u}_L \bigwedge_{i=1}^N [\bar{d}_i \vee u_{D_i,S}], & s=0 \\ 1-\bar{u}_L \bigwedge_{i=1}^N [\bar{d}_i \vee u_{D_i,S}], & s=1 \end{cases} \tag{15}$$

and

$$P(s^*|d_k, \mathrm{do}(\wedge_{i\neq k} D_i^* = 0), \mathrm{do}(u_L^* = 0)) = \begin{cases} \bar{d}_k \vee u_{D_k,S}, & s^*=0 \\ 1-\bar{d}_k \vee u_{D_k,S}, & s^*=1 \end{cases} \tag{16}$$

Taking the product of these functions gives the function $g_{s,s^*}(\underline{u}, \underline{d}, u_L) := P(s|d_k, \wedge_{i\neq k} d_i, u_L) \times P(s^*|d_k, \mathrm{do}(\wedge_{i\neq k} D_i^* = 0), \mathrm{do}(u_L^* = 0))$ where $\underline{u}$ denotes a given instantiation of the free latent variables $u_{D_1,S}, \ldots, u_{D_N,S}$.

$$g_{s,s^*}(\underline{u}, \underline{d}, u_L) = \begin{cases} \bar{u}_L \bigwedge\limits_{i=1}^N [\bar{d}_i \vee u_{D_i,S}], & s=s^*=0 \\ 0, \quad s=0, s^*=1 \\ [\bar{d}_k \vee u_{D_k,S}] \wedge [1-\bigwedge\limits_{i=1}^N [\bar{d}_i \vee u_{D_i,S}]], & s=1, s^*=0 \\ 1-\bar{d}_k \vee u_{D_k,S}, \quad s=1, s^*=1 \end{cases} \tag{17}$$

$$P(s, s^*|\underline{d}, \mathrm{do}(\wedge_{i\neq k} D_i^* = 0), \mathrm{do}(u_L^* = 0)) = \sum_{u_{D_1,S}} P(u_{D_1,S}) \cdots \sum_{u_{D_N,S}} P(u_{D_N,S}) \sum_{u_L} P(u_L) g_{s,s^*}(\underline{u}, \underline{d}, u_L) \tag{18}$$

$$= \begin{cases} \lambda_L \prod\limits_{i=1}^N \lambda_{D_i,S}^{d_i}, & s=s^*=0 \\ 0, \quad s=0, s^*=1 \\ \lambda_{D_k,s}^{d_k} - \lambda_L \prod\limits_{i=1}^N \lambda_{D_i,S}^{d_i}, & s=1, s^*=0 \\ (1-\lambda_{D_k,S})\delta(d_k-1), \quad s=1, s^*=1 \end{cases} \tag{19}$$

where we have used $\sum_{u_{D_i,S}} P(u_{D_i,S}) \bar{d}_i \vee u_{D_i,S} = P(u_{D_i,S}=1) + P(u_{D_i,S}=0)\bar{d}_i = P(u_{D_i,S}=1)^{d_i} = \lambda_{D_i,S}^{d_i}$, and $\sum_{u_{D_k,S}} P(u_{D_k,S})[1-\bar{d}_k \vee u_{D_k,S}] = (1-\lambda_{D_k,S})\delta(d_k-1)$, where $\delta(d_k-1)$ is 1 iff $D_k=1$ and 0 otherwise. $\lambda_L \prod\limits_{i=1}^N \lambda_{D_i,S}^{d_i}$ can immediately be identified as $P(s=0|\mathcal{D})$ by (11). $\lambda_{D_k,s}^{d_k} - \lambda_L \prod\limits_{i=1}^N \lambda_{D_i,S}^{d_i} = \lambda_{D_k,s}^{d_k}(1-\lambda_L \prod\limits_{i\neq k} \lambda_{D_i,S}^{d_i})$, and we can identify $\lambda_L \prod\limits_{i\neq k} \lambda_{D_i,S}^{d_i} = P(s=0| \wedge_{i\neq k} d_i, d_k=0)$. Therefore $\lambda_{D_k,s}^{d_k} - \lambda_L \prod\limits_{i=1}^N \lambda_{D_i,S}^{d_i} = \lambda_{D_k,s}^{d_k} P(s = 1|\wedge_{i\neq k} d_i, d_k=0)$. Finally, we can express this as $\lambda_{D_k,s}^{d_k} P(s^{\backslash k}=1|\wedge_{i\neq k} d_i, D_k=1)$, where $s^{\backslash k}$ is the instantiation of $S^{\backslash k}$ – which is the variable generated by removing any directed edge $D_k \to S$ (or equivalently, replacing $\lambda_{D_k,S}$ with 1).

□

Given our expression for the symptom CPT on the twin network, we now derive the expression for the expected sufficiency.

Theorem 1 *For noisy-OR networks described in supplementary note A.1-A.4, the expected sufficiency of disease* D_k *is given by*

$$\mathbb{E}_{\mathrm{suff}}(D_k, \mathcal{E}) = \frac{1}{P(\mathcal{S}_\pm|\mathcal{R})} \sum_{\mathcal{S}\subseteq\mathcal{S}_+} |\mathcal{S}_+ \setminus \mathcal{S}| P(\mathcal{S}_- = 0, \mathcal{S}^{\backslash k} = 1, D_k = 1|\mathcal{R}) \prod_{S\in\mathcal{S}_+\setminus\mathcal{S}} (1-\lambda_{D_k,S}) \prod_{S\in\mathcal{S}} \lambda_{D_k,S}$$

where $\mathcal{S}_\pm$ *denotes the positive and negative symptom evidence,* $\mathcal{R}$ *denotes the risk-factor evidence, and* $\mathcal{S}^{\backslash k}$ *denotes the set of symptoms* $\mathcal{S}$ *with all directed arrows from* D_k *to* $S \in \mathcal{S}$ *removed.*

Proof. Starting from the definition of the expected sufficiency

$$\mathbb{E}_{\text{suff}}(D_k, \mathcal{E}) := \sum_{\mathcal{S}'} |\mathcal{S}'_+| \, P(\mathcal{S}' | \mathcal{E}, \text{do}(\mathcal{D} \setminus D_k = 0), \text{do}(\mathcal{U}_L = 0)) \tag{20}$$

we must find expressions for all CPTs $P(\mathcal{S}'|\mathcal{E}, \text{do}(\mathcal{D} \setminus D_k = 0), \text{do}(\mathcal{U}_L = 0))$ where $|\mathcal{S}'_+| \neq 0$ (terms with $\mathcal{S}'_+ = \emptyset$ do not contribute to (20)). Let $\mathcal{S}^*_A = \{S^* \text{ s.t. } S \in \mathcal{S}_-, S^* \in \mathcal{S}'_-\}$ (symptoms that remain off following the counterfactual intervention), $\mathcal{S}^*_B = \{S^* \text{ s.t. } S \in \mathcal{S}_+, S^* \in \mathcal{S}'_+\}$ (symptoms that remain on following the counterfactual intervention), and $\mathcal{S}^*_C = \{S^* \text{ s.t. } S \in \mathcal{S}_+, S^* \in \mathcal{S}'_-\}$ (symptoms that are switched off by the counterfactual intervention). Lemma 1 implies that $P(S = 0, S^* = 1 | \underline{d}, \text{do}(\wedge_{i\neq k} D^*_i = 0), \text{do}(u^*_L = 0)) = 0$, and therefore these three cases are sufficient to characterise all possible counterfactual symptom states $\mathcal{S}'$. Therefore, to evaluate (20), we need only determine expressions for the following terms

$$P(S^*_A = 0, S^*_B = 1, S^*_C = 0 | \mathcal{S}_\pm, \mathcal{R}, \text{do}(\wedge_{i\neq k} D^*_i = 0), \text{do}(\mathcal{U}^*_L = 0)) \tag{21}$$

where $\mathcal{U}^*_L$ denotes the set of all counterfactual leak nodes for the symptoms $\mathcal{S}^*_A, \mathcal{S}^*_B, \mathcal{S}^*_C$. Note that we only perform counterfactual interventions, i.e. interventions on counterfactual variables. As the exogenous latents are shared by the factual and counterfactual graphs, $\mathcal{U}^*_L = U_L$, but we maintain the notation for clarity. First, note that

$$\begin{aligned} &P(S^*_A = 0, S^*_B = 1, S^*_C = 0 | \mathcal{S}_\pm, \mathcal{R}, \text{do}(\wedge_{i\neq k} D^*_i = 0), \text{do}(\mathcal{U}^*_L = 0)) \\ &= \frac{P(S^*_A = 0, S^*_B = 1, S^*_C = 0, \mathcal{S}_\pm | \mathcal{R}, \text{do}(\wedge_{i\neq k} D^*_i = 0), \text{do}(\mathcal{U}^*_L = 0))}{P(\mathcal{S}_\pm | \mathcal{R}, \text{do}(\wedge_{i\neq k} D^*_i = 0), \text{do}(\mathcal{U}^*_L = 0))} \\ &= \frac{P(S^*_A = 0, S^*_B = 1, S^*_C = 0, \mathcal{S}_\pm | \mathcal{R}, \text{do}(\wedge_{i\neq k} D^*_i = 0), \text{do}(\mathcal{U}^*_L = 0))}{P(\mathcal{S}_\pm | \mathcal{R})} \end{aligned}$$

Which follows from the fact that the factual symptoms $\mathcal{S}_\pm$ on the twin network [F] are conditionally independent from the counterfactual interventions $\text{do}(\wedge_{i\neq k} D^*_i = 0), \text{do}(\mathcal{U}^*_L = 0))$. To determine $Q = P(S^*_A = 0, S^*_B = 1, S^*_C = 0, \mathcal{S}_\pm | \mathcal{R}, \text{do}(\wedge_{i\neq k} D^*_i = 0), \text{do}(\mathcal{U}^*_L = 0))$, we express Q as a marginalization over the factual diseases which, together with the interventions on the counterfactual diseases and leak nodes, constitute a Markov blanket for each dual pair of symptoms

$$\begin{aligned} Q = &\sum_{d_1,\ldots,d_N} P(\wedge_{i\neq k} D_i = d_i, D_k = d_k | \mathcal{R}) \prod_{S \in \mathcal{S}_A} P(S^* = 0, S = 0 | \wedge_{i\neq k} D_i = d_i, D_k = d_k, \text{do}(\wedge_{i\neq k} D^*_i = 0), \text{do}(\mathcal{U}^*_L = 0)) \\ &\times \prod_{S \in \mathcal{S}_B} P(S^* = 1, S = 1 | \wedge_{i\neq k} D_i = d_i, D_k = d_k, \text{do}(\wedge_{i\neq k} D^*_i = 0), \text{do}(\mathcal{U}^*_L = 0)) \\ &\times \prod_{S \in \mathcal{S}_C} P(S^* = 0, S = 1 | \wedge_{i\neq k} D_i = d_i, D_k = d_k, \text{do}(\wedge_{i\neq k} D^*_i = 0), \text{do}(\mathcal{U}^*_L = 0)) \end{aligned} \tag{22}$$

Substituting in the CPT derived in Lemma 1 yields

$$\begin{aligned} Q = \sum_{d_1,\ldots,d_N} P(\wedge_{i\neq k} D_i = d_i, D_k = d_k | \mathcal{R}) &\prod_{S \in \mathcal{S}_A} P(s = 0 | \wedge_{i=1}^N d_i) \prod_{S \in \mathcal{S}_B} (1 - \lambda_{D_k,S}) \delta(d_k - 1) \\ &\times \prod_{S \in \mathcal{S}_C} \lambda^{d_k}_{D_k,s} P(s^{\setminus k} = 1 | \wedge_{i\neq k} d_i, D_k = 1) \end{aligned} \tag{23}$$

The only terms in (20) with $|\mathcal{S}'_+| \neq 0$ have $\mathcal{S}_B \neq \emptyset$, therefore the term $\delta(d_k - 1)$ is present, and Q simplifies to

$$\begin{aligned} Q = \sum_{d_i \forall i \neq k} P(\wedge_{i\neq k} D_i = d_i, D_k = 1 | \mathcal{R}) &\prod_{S \in \mathcal{S}_A} P(s = 0 | \wedge_{i\neq k}^N d_i, D_k = 1) \prod_{S \in \mathcal{S}_B} (1 - \lambda_{D_k,S}) \\ &\times \prod_{S \in \mathcal{S}_C} \lambda_{D_k,s} P(s^{\setminus k} = 1 | \wedge_{i\neq k} d_i, D_k = 1) \end{aligned} \tag{24}$$

$$= P(S_A = 0, S^{\setminus k}_C = 1, D_k = 1 | \mathcal{R}) \prod_{S \in S_B} (1 - \lambda_{D_k,S}) \prod_{S \in S_C} \lambda_{D_k,S} \tag{25}$$

where in the last line we have performed the marginalization over $d_i \ \forall\ i \neq k$. Finally, $\mathcal{S}'_+ = \mathcal{S}^*_B = \mathcal{S}_+ \setminus \mathcal{S}_C$, and so $|\mathcal{S}'_+| = |\mathcal{S}_+| - |\mathcal{S}_C|$, and the expected expected sufficiency is

$$\mathbb{E}_{\text{suff}}(D_k, \mathcal{E}) = \frac{1}{P(\mathcal{S}_\pm|\mathcal{R})} \sum_{\mathcal{S}\subseteq\mathcal{S}_+} (|\mathcal{S}_+| - |\mathcal{S}|)\, P(\mathcal{S}_- = 0, \mathcal{S}^{\setminus k} = 1, D_k = 1|\mathcal{R}) \prod_{S\in\mathcal{S}_+\setminus\mathcal{S}} (1-\lambda_{D_k,S}) \prod_{S\in\mathcal{S}} \lambda_{D_k,S} \tag{26}$$

where we have dropped the subscript C from $\mathcal{S}_C$.

□

Given our expression for the expected sufficiency, we now derive a simplified expression that is very similar to the posterior $P(D_k = 1|\mathcal{R}, \mathcal{S}_\pm)$.

Theorem 2 (Simplified expected sufficiency)**.**

$$\mathbb{E}_{suff}(D_k, \mathcal{E}) = \frac{1}{P(\mathcal{S}_\pm|\mathcal{R})} \sum_{\mathcal{Z}\subseteq\mathcal{S}_+} (-1)^{|\mathcal{Z}|} P(\mathcal{S}_- = 0, \mathcal{Z} = 0, D_k = 1|\mathcal{R}) \times \tau(k, \mathcal{Z}) \tag{27}$$

where

$$\tau(k, \mathcal{Z}) = \sum_{S\in\mathcal{S}_+\setminus\mathcal{Z}} (1-\lambda_{D_k,S}) \tag{28}$$

Proof. Starting with the expected sufficiency given in Theorem 2, we can perform the change of variables $\mathcal{X} = \mathcal{S}_+ \setminus \mathcal{S}$ to give

$$\mathbb{E}_{\text{suff}}(D_k, \mathcal{E}) = \frac{1}{P(\mathcal{S}_\pm|\mathcal{R})} \sum_{\mathcal{X}\subseteq\mathcal{S}_+} |X| \prod_{S\in\mathcal{X}} (1-\lambda_{D_k,S}) \prod_{S\in\mathcal{S}_+\setminus\mathcal{X}} \lambda_{D_k,S}\, P(\mathcal{S}_- = 0, (\mathcal{S}_+ \setminus \mathcal{X})^{\setminus k} = 1, D_k = 1|\mathcal{R}) \tag{29}$$

$$= \frac{1}{P(\mathcal{S}_\pm|\mathcal{R})} \sum_{\mathcal{X}\subseteq\mathcal{S}_+} |\mathcal{X}| \prod_{S\in\mathcal{X}} (1-\lambda_{D_k,S}) \prod_{S\in\mathcal{S}_+\setminus\mathcal{X}} \lambda_{D_k,S} \sum_{\mathcal{Z}\subseteq\mathcal{S}_+\setminus\mathcal{X}} (-1)^{|\mathcal{Z}|} P(\mathcal{S}_- = 0, \mathcal{Z}^{\setminus k} = 0, D_k = 1|\mathcal{R}) \tag{30}$$

where in the last line we apply the inclusion-exclusion principle to decompose an arbitrary joint state over Bernoulli variables $P(\mathcal{A} = 0, \mathcal{B} = 1)$ as a sum over the powerset of the variables $\mathcal{B}$ in terms of marginals where all variables are instantiated to 0,

$$P(\mathcal{A} = 0, \mathcal{B} = 1) = \sum_{\mathcal{C}\subseteq\mathcal{B}} (-1)^{|C|} P(\mathcal{A} = 0, \mathcal{C} = 0) \tag{31}$$

By the definition of noisy-or (7) we have that

$$\begin{aligned}
P(\mathcal{S}_- = 0, &\mathcal{Z}^{\setminus k} = 0, D_k = 1|\mathcal{R}) \\
&= \sum_{d_i, i\neq k} P(\mathcal{S}_- = 0, \mathcal{Z}^{\setminus k} = 0, D_k = 1, \wedge_{i\neq k}^N D_i = d_i|\mathcal{R}) \\
&= \sum_{d_i, i\neq k} \prod_{S\in\mathcal{S}_-} P(S = 0|D_k = 1, \wedge_{i\neq k}^N D_i = d_i) \prod_{S\in\mathcal{Z}} P(S^{\setminus k} = 0|D_k = 1, \wedge_{i\neq k}^N D_i = d_i) P(D_k = 1, \wedge_{i\neq k}^N D_i = d_i|\mathcal{R}) \\
&= \sum_{d_i, i\neq k} \prod_{S\in\mathcal{S}_-} P(S = 0|D_k = 1, \wedge_{i\neq k}^N D_i = d_i) \prod_{S\in\mathcal{Z}} \frac{P(S = 0|D_k = 1, \wedge_{i\neq k}^N D_i = d_i)}{\lambda_{D_k,S}} P(D_k = 1, \wedge_{i\neq k}^N D_i = d_i|\mathcal{R}) \\
&= \frac{P(\mathcal{S}_- = 0, \mathcal{Z} = 0, D_k = 1|\mathcal{R})}{\prod_{S\in\mathcal{Z}} \lambda_{D_k,S}}
\end{aligned} \tag{32}$$

Therefore we can replace the graph operation represented by $\setminus k$ by dividing the CPT by the product $\prod_{S\in\mathcal{Z}} \lambda_{D_k,S}$. This allows $\mathbb{E}_{\text{suff}}$ to be expressed as

$$\mathbb{E}_{\text{suff}}(D_k, \mathcal{E}) = \frac{1}{P(\mathcal{S}_\pm|\mathcal{R})} \sum_{\mathcal{X}\subseteq\mathcal{S}_+} |\mathcal{X}| \prod_{S\in\mathcal{X}} (1-\lambda_{D_k,S}) \prod_{S\in\mathcal{S}_+\setminus\mathcal{X}} \lambda_{D_k,S} \sum_{\mathcal{Z}\subseteq\mathcal{S}_+\setminus\mathcal{X}} (-1)^{|\mathcal{Z}|} P(\mathcal{S}_- = 0, \mathcal{Z} = 0, D_k = 1|\mathcal{R}) \frac{1}{\prod_{S\in\mathcal{Z}} \lambda_{D_k,S}} \tag{33}$$

We now aggregate the terms in the power sum that yield the same marginal on the symptoms (e.g. for fixed $\mathcal{Z}$). Every $\mathcal{X} \in \mathcal{S}_+ \setminus \mathcal{Z}$ yields a single marginal $P(\mathcal{S}_- = 0, \mathcal{Z} = 0, D_k = 1|\mathcal{R})$ and therefore if we express (33) as a sum in terms of $\mathcal{Z}$, where each term $P(\mathcal{S}_- = 0, \mathcal{Z} = 0, D_k = 1|\mathcal{R})$ aggregates the a coefficient $K_\mathcal{Z}$ of the form $\mathbb{E}_{\text{suff}}(D_k, \mathcal{E}) = \sum_{\mathcal{Z}\subseteq\mathcal{S}_+} K_\mathcal{Z} P(\mathcal{S}_- = 0, \mathcal{Z} = 0, D_k = 1|\mathcal{R})$ where

$$\begin{aligned}
K_\mathcal{Z} &= \frac{(-1)^{|\mathcal{Z}|}}{P(\mathcal{S}_\pm|\mathcal{R})} \frac{1}{\prod\limits_{S\in\mathcal{Z}} \lambda_{D_k,S}} \sum_{\mathcal{X}\subseteq\mathcal{S}_+\setminus\mathcal{Z}} |\mathcal{X}| \prod_{S\in\mathcal{X}} (1-\lambda_{D_k,S}) \prod_{S\in\mathcal{S}_+\setminus\mathcal{X}} \lambda_{D_k,S} \\
&= \frac{(-1)^{|\mathcal{Z}|}}{P(\mathcal{S}_\pm|\mathcal{R})} \frac{1}{\prod\limits_{S\in\mathcal{Z}} \lambda_{D_k,S}} \sum_{\mathcal{X}\subseteq\mathcal{A}} |\mathcal{X}| \prod_{S\in\mathcal{X}} (1-\lambda_{D_k,S}) \prod_{S\in\mathcal{A}\setminus\mathcal{X}} \lambda_{D_k,S} \prod_{S\in\mathcal{Z}} \lambda_{D_k,S} \\
&= \frac{(-1)^{|\mathcal{Z}|}}{P(\mathcal{S}_\pm|\mathcal{R})} \sum_{\mathcal{X}\subseteq\mathcal{A}} |\mathcal{X}| \prod_{S\in\mathcal{X}} (1-\lambda_{D_k,S}) \prod_{S\in\mathcal{A}\setminus\mathcal{X}} \lambda_{D_k,S} \qquad (34)
\end{aligned}$$

where $\mathcal{A} = \mathcal{S}_+ \setminus \mathcal{Z}$. This can be further simplified using the identity

$$\sum_{A\subseteq B} |A| \prod_{a\in A} (1-a) \prod_{a'\in B\setminus A} a' = |B| - \sum_{a\in B} a = \sum_{a\in B} (1-a) \qquad (35)$$

which we now prove iteratively. First, consider the function $S(\mathcal{B}) := \sum\limits_{\mathcal{A}\subseteq\mathcal{B}} \prod\limits_{a\in\mathcal{A}} (1-a) \prod\limits_{a'\in\mathcal{B}\setminus\mathcal{A}} a'$. Now, consider $S(\mathcal{B} + \{c\})$. This function can be divided into two sums, one where $c \in \mathcal{A}$ and the other where $c \notin \mathcal{A}$. Therefore

$$S(\mathcal{B} + \{c\}) = \sum_{\mathcal{A}\subseteq\mathcal{B}} \prod_{a\in\mathcal{A}} (1-a) \prod_{a'\in\mathcal{B}\setminus\mathcal{A}} a'c + \sum_{\mathcal{A}\subseteq\mathcal{B}} \prod_{a\in\mathcal{A}} (1-a) \prod_{a'\in\mathcal{B}\setminus\mathcal{A}} a'(1-c) = S(\mathcal{B}) \qquad (36)$$

Starting with the empty set, $S(\emptyset) = 1$, it follows that $S(\mathcal{B}) = 1 \;\forall$ countable sets $\mathcal{B}$. Next, consider the function $G(\mathcal{B}) := \sum\limits_{\mathcal{A}\subseteq\mathcal{B}} |\mathcal{A}| \prod\limits_{a\in\mathcal{A}} (1-a) \prod\limits_{a'\in\mathcal{B}\setminus\mathcal{A}} a'$, which is the form of the sum we wish to compute in (34). Proceeding as before, we have

$$\begin{aligned}
G(\mathcal{B} + \{c\}) &= \sum_{\mathcal{A}\subseteq B} |\mathcal{A}| \prod_{a\in\mathcal{A}} (1-a) \prod_{a'\in\mathcal{B}\setminus\mathcal{A}} a'c + \sum_{\mathcal{A}\subseteq\mathcal{B}} (|\mathcal{A}|+1) \prod_{a\in\mathcal{A}} (1-a) \prod_{a'\in\mathcal{B}\setminus\mathcal{A}} a'(1-c) \\
&= cG(\mathcal{B}) + (1-c)G(\mathcal{B}) + (1-c)S(\mathcal{B})
\end{aligned}$$

Using $S(\mathcal{B}) = 1$ we arive at the recursive formula $G(\mathcal{B} + \{c\}) = G(\mathcal{B}) + (1-c)$. Starting with $G(\emptyset) = 0$, and building the set $\mathcal{B}$ by recursively adding elements c to the set, we arrive at the identity

$$G(\mathcal{B}) = |\mathcal{B}| - \sum_{a\in\mathcal{B}} a \qquad (37)$$

Using (37) we can simplify the coefficient (34)

$$\frac{(-1)^{|\mathcal{Z}|}}{P(\mathcal{S}_\pm|\mathcal{R})} \sum_{\mathcal{X}\subseteq\mathcal{S}_+\setminus\mathcal{Z}} |\mathcal{X}| \prod_{S\in\mathcal{X}} (1-\lambda_{D_k,S}) \prod_{S\in(\mathcal{S}_+\setminus\mathcal{Z})\setminus\mathcal{X}} \lambda_{D_k,S} = \frac{(-1)^{|\mathcal{Z}|}}{P(\mathcal{S}_\pm|\mathcal{R})} \sum_{S\in S_+\setminus\mathcal{Z}} (1-\lambda_{D_k,S}) \qquad (38)$$

Rearranging (33) as a summation over $\mathcal{Z}$ substituting in (38) gives

$$\mathbb{E}_{\text{suff}}(D_k, \mathcal{E}) = \frac{1}{P(\mathcal{S}_\pm|\mathcal{R})} \sum_{\mathcal{Z}\subseteq\mathcal{S}_+} (-1)^{|\mathcal{Z}|} P(\mathcal{S}_- = 0, \mathcal{Z} = 0, D_k = 1|\mathcal{R}) \left(\sum_{S\in\mathcal{S}_+\setminus\mathcal{Z}} (1-\lambda_{D_k,S}) \right) \qquad (39)$$

which can be expressed as

$$\mathbb{E}_{\text{suff}}(D_k, \mathcal{E}) = \frac{1}{P(\mathcal{S}_\pm|\mathcal{R})} \sum_{\mathcal{Z}\subseteq\mathcal{S}_+} (-1)^{|\mathcal{Z}|} P(\mathcal{S}_- = 0, \mathcal{Z} = 0, D_k = 1|\mathcal{R}) \times \tau(k, \mathcal{Z}) \qquad (40)$$

where

$$\tau(k,\mathcal{Z}) = \sum_{S\in\mathcal{S}_+\setminus\mathcal{Z}} (1-\lambda_{D_k,S}) \tag{41}$$

Note that if we fix $\tau(k,\mathcal{Z}) = 1\ \forall\mathcal{Z}$, we recover $\sum_{\mathcal{Z}\subseteq\mathcal{S}_+} (-1)^{|\mathcal{Z}|} P(\mathcal{S}_- = 0, \mathcal{Z} = 0, D_k = 1|\mathcal{R})/P(\mathcal{S}_\pm|\mathcal{R}) = P(\mathcal{S}_\pm, D_k = 1|\mathcal{R})/P(\mathcal{S}_\pm|\mathcal{R}) = P(D_k = 1|\mathcal{E})$, which is the standard posterior of disease D_k under evidence $\mathcal{E} = \mathcal{R}\cap\mathcal{S}_\pm$ (this follows from the inclusion-exclusion principle, and can be easily checked by applying marginalization to express $P(\mathcal{S}_\pm, D_k = 1|\mathcal{R})$ in terms of marginals where all symptoms are instantiated as 0). Note that (40) can be seen as a counterfactual correction to the quickscore algorithm in [10] (although we do not assume independence of diseases as the authors of [10] do).

□

Supplementary note 5: properties of the expected sufficiency

In this supplementary note, we show that the expected sufficiency (42) obeys our four postulates, including an additional postulate of sufficiency which is obeyed by the expected sufficiency.

Theorem 3 (Diagnostic properties of expected sufficiency). *1.* consistency. $\mathbb{E}_{suff}(D_k,\mathcal{E}) \propto P(D_k = 1|\mathcal{E})$

2. causality. *If* $\nexists\ S \in \mathsf{Dec}(D_k) \cap \mathcal{S}_+ \implies \mathbb{E}_{suff}(D_k,\mathcal{E}) = 0$

3. simplicity. $|\mathbb{E}_{suff}(D_k,\mathcal{E})| \leq |\mathcal{S}_+ \cap \mathsf{Dec}(D_k)|$

4. sufficiency. $\mathbb{E}_{suff}(D_i \wedge D_j,\mathcal{E}) > 0 \implies \mathbb{E}_{suff}(D_i,\mathcal{E}) > 0$ *and* $\mathbb{E}_{suff}(D_j,\mathcal{E}) > 0$

The expected sufficiency satisfies the following four properties,

Proof. Postulate 1 dictates that the measure should be proportional to the posterior probability of the diseases. Postulate 2 states that if the disease has no causal effect on the symptoms presented then it is a poor diagnosis and should be discarded. Postulate 3 states that the (tight) upper bound of the measure for a given disease (in the sense that there exists some disease model that achieves this upper bound – namely deterministic models) is the number of positive symptoms that the disease can explain. This allows us to differentiate between diseases that are equally likely causes, but where one can explain more symptoms than another. Postulate 4 states that if it is possible that D_k is causing at least one symptom, then the measure should be strictly greater than 0. Starting from the definition of the expected sufficiency

$$\mathbb{E}_{\text{suff}}(D_k,\mathcal{E}) := \sum_{\mathcal{S}'} |\mathcal{S}'_+|\ P(\mathcal{S}'|\mathcal{E}, \text{do}(\mathcal{D}\setminus D_k = 0), \text{do}(\mathcal{U}_L = 0)) \tag{42}$$

given the conditional independence structure of the twin network [F], we can express the counterfactual symptom marginals as

$$P(\mathcal{S}'|\mathcal{E}, \text{do}(\mathcal{D}\setminus D_k = 0), \text{do}(\mathcal{U}_L = 0)) \tag{43}$$
$$= \sum_{d_k} \prod_{S^*\in\mathcal{S}'} P(S^*|\mathcal{E}, \text{do}(\mathcal{D}^*\setminus D_k = 0), \text{do}(\mathcal{U}^*_L = 0), d_k) P(d_k|\mathcal{E}, \text{do}(\mathcal{D}^*\setminus D_k = 0), \text{do}(\mathcal{U}^*_L = 0)) \tag{44}$$
$$= \sum_{d_k} \prod_{S^*\in\mathcal{S}'} P(S^*|\mathcal{E}, \text{do}(\mathcal{D}^*\setminus D_k = 0), \text{do}(\mathcal{U}^*_L = 0), d_k) P(d_k|\mathcal{E}) \tag{45}$$

If $D_k = 1$, then do the the counterfactual interventions the counterfactual states have all parents (including leaks) instantiated to 0, which implies that $\mathcal{S}'_+ = \emptyset$ by (2). Hence this case never contributes to the expected sufficiency as the expectation is over $|\mathcal{S}'_+|$. For $D_k = 1$, we recover that $P(\mathcal{S}'|\mathcal{E}, \text{do}(\mathcal{D}\setminus D_k = 0), \text{do}(\mathcal{U}_L = 0)) \propto P(D_k = 1|\mathcal{E})$ and therefore $\mathbb{E}_{\text{suff}}(D_k,\mathcal{E}) \propto P(D_k = 1|\mathcal{E})$. For postulate 2, if there are no symptoms that are descendants of D_k, then $\mathbb{E}_{\text{suff}}(D_k,\mathcal{E}) = 0$. This follows immediately from the fact that if D_k is not an ancestor of any of the symptoms, then all counterfactual symptoms have all parents instantiated as 0 and $\mathcal{S}'_+ = \emptyset$. For postulate 4, we can only prove this property under additional assumptions about our disease model (see supplementary note 2 for noisy-and counter example). First, note that $\mathbb{E}_{\text{suff}}(D_k,\mathcal{E})$ is a convex sum with positive semi-definite coefficients $|\mathcal{S}'_+|$. If there is a single positively evidenced symptom that is a descendent of D_k, and D_k has a positive causal influence on that child, and our disease model permits that every disease be capable of causing its associated symptoms in isolation, i.e. $P(S = 1|\text{only})(D_k = 1)) > 0$ for $S \in \mathsf{Dec}(D_k)$, then it is simple to check that $P(S^* = 1|\mathcal{E}, \text{do}(\mathcal{D}^*\setminus D_k = 0), \text{do}(\mathcal{U}^*_L = 0), d_k = 1) > 0$ and so $E_{\text{suff}}(D_k,\mathcal{E}) > 0$. □

Supplementary note 6: expected disablement

In this supplementary note we turn our attention to our second diagnostic measure − the expected disablement. This measure is closer to typical treatment measures, such as the effect of treatment on the treated [24]. We use our twin diagnostic network outlined in supplementary note 3 figure [E] (shown below) to simulating counterfactual treatments. We focus on the simplest case of single disease interventions, and propose a simple ranking measure whereby the best treatments are those that get rid of the most symptoms.

Definition 3 *The expected disablement of disease D_k determines the number of positive symptoms that we would expect to switch off if we intervened to turn off D_k,*

$$\mathbb{E}_{\text{dis}}(D_k, \mathcal{E}) := \sum_{\mathcal{S}'} \left|\mathcal{S}_+ \setminus \mathcal{S}'_+\right| P(\mathcal{S}'|\mathcal{E}, \text{do}(D_k = 0)) \tag{46}$$

where $\mathcal{E}$ is the factual evidence and $\mathcal{S}_+$ is the set of factual positively evidenced symptoms. The expectation is calculated over all possible counterfactual symptom evidence states $\mathcal{S}'$ and $\mathcal{S}'_+$ denotes the positively evidenced symptoms in the counterfactual symptom evidence state. do$(D_k = 0)$ denotes the counterfactual intervention setting $D_k \to 0$.

Decisions about which treatment to select for a patient generally take into account variables such as cost and cruelty. These variables can be simply included in the treatment measure. For example, the cruelty of specific symptoms can be included in the expectation (46) by weighting each positive symptom accordingly. The cost of treating a specific disease is included simply by multiplying (46) by a cost weight, and likewise for including the probability of the intervention succeeding. For now, we focus on computing the counterfactual probabilities, which we can then use to construct arbitrarily weighted expectations.

To calculate (46), note that the only CPTs that differ from the original noisy-OR SCM are those for unmerged dual symptom nodes (i.e. children of the intervention node D_k). The disease layer forms a Markov blanket for the symptoms layer, d-separating dual symptom pairs from each other. Therefore we derive the CPT for dual symptoms and their parent diseases.

[E]

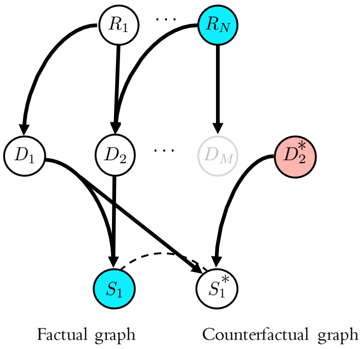

Supplementary Figure 4: Final twin network for expected disablement

Lemma 2. *For a given symptom S and its counterfactual dual S^*, with parent diseases $\mathcal{D}$ and under the counterfactual intervention do$(D_k^* = 0)$, the joint conditional distribution on the twin network is given by*

$$P(s, s^* \mid \wedge_i D_i = d_i, do(D_k^* = 0)) = \begin{cases} P(s = 0 \mid \wedge_i D_i = d_i) \quad \text{if } s = s^* = 0 \\ 0 \quad \text{if } s = 0, s^* = 1 \\ \left(\frac{1}{\lambda_{D_k,S}} - 1\right) P(s = 0 \mid \wedge_{i \neq k} D_i = d_i, D_k = 1)\delta(d_k - 1) \quad \text{if } s = 1, s^* = 0 \text{ and } \lambda_{D_k,S} \neq 0 \\ P(s^{\setminus k} = 0 \mid \wedge_{i \neq k} D_i = d_i, D_k = 1)\delta(d_k - 1) \quad \text{if } s = 1, s^* = 0 \text{ and } \lambda_{D_k,S} = 0 \\ P(s^{\setminus k} = 1 \mid \wedge_{i \neq k} D_i = d_i, D_k = 1) \quad \text{if } s = 1, s^* = 1 \end{cases}$$

where $\delta(d_k - 1) = 1$ if $D_k = 1$ else 0.

Proof. First note that for this marginal distribution the intervention do$(D_k^* = 0)$ is equivalent to setting the evidence $D_k^* = 0$ as we specify the full Markov blanket of (s, s^*). Let $\mathcal{D}_{\setminus k}$ denote the set of parents of (s, s^*) not including the intervention node D_k^* or its dual D_k. We wish to compute the conditional probability

$$P(s, s^* \mid \wedge_{i \neq k} D_i = d_i, D_k = d_k) = \sum_{\underline{u}_s} p(\underline{u}_s) P(s | \wedge_{i \neq k} D_i = d_i, D_k = d_k, u_s) P(s^* | \wedge_{i \neq k} D_i = d_i, D_k^* = 0, u_s) \tag{47}$$

where $p(\underline{u}_s)$ is the product distribution over all exogenous noise terms for S including the leak term. We proceed as before by expressing this as a marginalization over the CPT of the dual states, $P(s=0, s^*=0 \mid \wedge_{i\neq k} D_i = d_i, D_k^* = 0, D_k)$, $P(s=0 \mid \wedge_{i\neq k} D_i = d_i, D_k^* = 0, D_k = d_k)$ and $P(s^*=0 \mid \wedge_{i\neq k} D_i = d_i, D_k^* = 0, D_k = d_k)$. For $s_i = 0$, the generative functions are given by

$$P(s=0 \mid \mathsf{Pa}(S), u_s) = u_L \bigwedge_{D_i \in \mathsf{Pa}(S)} (\bar{d}_i \vee u_{D_i,S}) \tag{48}$$

First we compute the joint state.

$$\begin{aligned}
&P(s=0| \wedge_{i\neq k} D_i = d_i, D_k = d_k, u_s) P(s^* = 0 | \wedge_{i\neq k} D_i = d_i, D_k^* = d_k^*, u_s) \\
&= u_L \wedge u_L \bigwedge_{D_i \in \mathcal{D}_{\setminus k}} (u_{D_i,S} \vee \bar{d}_i) \bigwedge_{D_j \in \mathcal{D}_{\setminus k}} (u_{D_j,S} \vee \bar{d}_j) \wedge \left[u_{D_k,S} \vee \bar{d}_k\right] \wedge \left[u_{D_k,S} \vee \bar{d}_k^*\right] \\
&= u_L \bigwedge_{D_i \in \mathcal{D}_{\setminus k}} (u_{D_i,S} \vee \bar{d}_i) \left[u_{D_k,S} \vee \left(\bar{d}_k^* \wedge \bar{d}_k\right)\right]
\end{aligned}$$

Where we have used the Boolean identities $a \wedge a = a$ and $a \vee (b \wedge c) = (a \vee b) \wedge (a \vee c)$. Therefore

$$\begin{aligned}
P(s=0, s^*=0| \wedge_{i\neq k} D_i = d_i, D_k = d_k, D_k^* = d_k^*) &= \sum_{\underline{u}_s} p(\underline{u}_s) P(s=0|\mathcal{D}_{\setminus k}, D_k, u_s) P(s^*=0|\mathcal{D}_{\setminus k}, D_k^*, u_s) \\
&= \lambda_{L_s} \left[\lambda_{D_k,S} (d_k \vee d_k^*) + \bar{d}_k \wedge \bar{d}_k^*\right] \prod_{D_i \in \mathcal{D}_{\setminus k}} \left[\lambda_{D_i,S} d_i + \bar{d}_i\right]
\end{aligned}$$

Next, we calculate the single-symptom conditionals

$$\begin{aligned}
P(s=0 \mid \wedge_{i\neq k} D_i = d_i, D_k = d_k) &= \sum_{\underline{u}_s} p(\underline{u}_s) P(s=0|\mathcal{D}_{\setminus k}, D_k, u_s) \\
&= \sum_{u_{L_S}} P(u_{L_s}) u_{L_s} \prod_{D_i \in \mathcal{D}} \sum_{u_{D_i,S}} P(u_{D_i,S}) u_{D_i,S} \vee \bar{d}_i \\
&= P(u_{L_s} = 1) \prod_{D_i \in \mathcal{D}} \sum_{u_{D_i,S}} \left[P(u_{D_i,S} = 1) + P(u_{D_i,S} = 0) \bar{d}_i\right] \\
&= \lambda_{L_s} \prod_{D_i \in \mathcal{D}} \left[\lambda_{D_i,S} d_i + \bar{d}_i\right]
\end{aligned} \tag{49}$$

and similar for $P(s^* = 0 \mid \wedge_{i\neq k} D_i = d_i, D_k^* = d_k^*)$. Note that $\lambda x + \bar{x} = \lambda^x$. We can now express the joint cpd over dual symptom pairs, using the identities $P(s=0, s^*=1 \mid X) = P(s=0 \mid X) - P(s=0, s^*=0 \mid X)$, $P(s=1, s^*=0 \mid X) = P(s^*=0 \mid X) - P(s=0, s^*=0 \mid X)$ and $P(s=1, s^*=1 \mid X) = 1 - P(s=0 \mid X) - P(s^* = 0 \mid X) + P(s=0, s^*=0 \mid X)$ for arbitrary conditional X.

$$P(s, s^* | \wedge_{i\neq k} D_i = d_i, D_k = d_k, D_k^* = d_k^*) = \begin{cases}
\lambda_{L_s} \lambda_{D_k,S}^{d_k \vee d_k^*} \prod_{D_i \in \mathcal{D}_{\setminus k}} \lambda_{D_i,S}^{d_i} & \text{if } s = s^* = 0 \\
\lambda_{L_s} \left[\lambda_{D_k,S}^{d_k} - \lambda_{D_k,S}^{d_k \vee d_k^*}\right] \prod_{D_i \in \mathcal{D}_{\setminus k}} \lambda_{D_i,S}^{d_i} & \text{if } s = 0, s^* = 1 \\
\lambda_{L_s} \left[\lambda_{D_k,S}^{d_k^*} - \lambda_{D_k,S}^{d_k \vee d_k^*}\right] \prod_{D_i \in \mathcal{D}_{\setminus k}} \lambda_{D_i,S}^{d_i} & \text{if } s = 1, s^* = 0 \\
1 - \lambda_{L_s} \left[\lambda_{D_k,S}^{d_k} + \lambda_{D_k,S}^{d_k^*} - \lambda_{D_k,S}^{d_k \vee d_k^*}\right] \prod_{D_i \in \mathcal{D}_{\setminus k}} \lambda_{D_i,S}^{d_i} & \text{if } s = s^* = 1
\end{cases}$$

As we are always intervening to switch off diseases, $D_k^* = 0$, then $d_k \vee d_k^* = d_k$ and

$$\lambda_{D_k,S}^{d_k} - \lambda_{D_k,S}^{d_k \vee d_k^*} = 0 \tag{50}$$

and therefore $P(s=0, s^*=1 | \wedge_{i\neq k} D_i = d_i, D_k = d_k, D_k^* = 0) = 0$ as expected (switching off a disease will never switch on a symptom). This simplifies our expression for the conditional distribution to

$$P(s, s^*|\wedge_{i\neq k} D_i = d_i, D_k = d_k, D_k^* = 0) = \begin{cases} \lambda_{L_s} \lambda_{D_k,S}^{d_k} \prod\limits_{D_i \in \mathcal{D}_{\setminus k}} \lambda_{D_i,S}^{d_i} & \text{if } s = s^* = 0 \\ 0 \quad \text{if } s = 0, s^* = 1 \\ \lambda_{L_s} \left[1 - \lambda_{D_k,S}^{d_k}\right] \prod\limits_{D_i \in \mathcal{D}_{\setminus k}} \lambda_{D_i,S}^{d_i} & \text{if } s = 1, s^* = 0 \\ 1 - \lambda_{L_s} \prod\limits_{D_i \in \mathcal{D}_{\setminus k}} \lambda_{D_i,S}^{d_i} & \text{if } s = s^* = 1 \end{cases} \tag{51}$$

This then simplifies using (49) to

$$P(s, s^*|\wedge_{i\neq k} D_i = d_i, D_k = d_k, D_k^* = 0) = \begin{cases} P(s = 0|\wedge_i D_i = d_i) \quad \text{if } s = s^* = 0 \\ 0 \quad \text{if } s = 0, s^* = 1 \\ P(s = 0|\wedge_{i\neq k} D_i = d_i, D_k = 0) - P(s = 0|\wedge_{i\neq k} D_i = d_i, D_k = d_k) \text{ if } s = 1, s^* = \text{[illegible]} \\ P(s = 1|\wedge_{i\neq k} D_i = d_i, D_k = 0) \quad \text{if } s = s^* = 1 \end{cases} \tag{52}$$

We have arrived at expressions for the CPT's over dual symptoms in terms of CPT's on the factual graph, and hence our conterfactual query can be computed on the factual graph alone. The third term in (52), $P(s = 0|\wedge_{i\neq k} D_i = d_i, D_k = 0) - P(s = 0|\wedge_{i\neq k} D_i = d_, D_k = d_k)$, equals zero unless $D_k = 1$. Using the definition of noisy-OR (7) to give

$$P(s = 0|\wedge_{i\neq k} D_i = d_i, D_k = 0) = \frac{1}{\lambda_{D_k,S}} P(s = 0|\wedge_{i\neq k} D_i = d_i, D_k = 1) \tag{53}$$

in the case that $\lambda_{D_k,S} > 0$, we recover

$$P(s = 0|\wedge_{i\neq k} D_i = d_i, D_k = 0) - P(s = 0|\wedge_{i\neq k} D_i = d_i, D_k = d_k) = \left(\frac{1}{\lambda_{D_k,S}} - 1\right) P(s = 0\,|\wedge_{i\neq k} D_i = d_i, D_k = 1)\delta(d_k - 1) \tag{54}$$

where d_k is the instantiation of D_k on the factual graph. The term $\delta(d_k - 1)$ is equivalent to fixing the observation $D_k = 1$ on the factual graph. If $\lambda_{D_k,S} = 0$ then

$$\lambda_{L_s} \left[1 - \lambda_{D_k,S}^{d_k}\right] \prod_{D_i \in \mathcal{D}_{\setminus k}} \lambda_{D_i,S}^{d_i} = \lambda_{L_s} \prod_{D_i \in \mathcal{D}_{\setminus k}} \lambda_{D_i,S}^{d_i} \delta(d_k - 1) \tag{55}$$

which is equivalent to $P(s^{\setminus k} = 0|\wedge_{i\neq k} D_i = d_i, D_k = 1)\delta(d_k - 1)$

Finally, from the definition of the noisy-OR CPT (2),

$$P(s = 1|\wedge_{i\neq k} D_i = d_i, D_k = 0) = P(s^{\setminus k} = 1|\wedge_{i\neq k} D_i = d_i, D_k = 1) \tag{56}$$

□

Lemma 52 allows us to express the expected disablement in terms of factual probabilities. As we have seen, the intervention $\text{do}(D_k^* = 0)$ can never result in counterfactual symptoms that are on, when their dual factual symptoms are off, so we need only enumerate over counterfactual symptoms states where $\mathcal{S}'_+ \subseteq \mathcal{S}_+$ as these are the only counterfactual states with non-zero weight. From this it also follows that for all $s \in \mathcal{S}_- \implies s^* \in \mathcal{S}'_-$. The counterfactual CPT in (46) is represented on the twin network [F] as

$$P(\mathcal{S}'_+, \mathcal{S}'_-|\mathcal{E}, \text{do}(D_k^* = 0)) = P(\mathcal{S}'_+, \mathcal{S}'_-|\mathcal{S}_+, \mathcal{S}_-, \mathcal{R}, \text{do}(D_k^* = 0)) \tag{57}$$

Theorem 4 (Simplified noisy-OR expected disablement)**.** *For the noisy-OR networks described in supplementary note 2, the expected disablement of disease D_k is given by*

$$\mathbb{E}_{dis}(D_k, \mathcal{E}) = \frac{1}{P(\mathcal{S}_+, \mathcal{S}_-|\mathcal{R})} \sum_{\mathcal{Z} \subseteq \mathcal{S}_+} (-1)^{|\mathcal{Z}|} P(\mathcal{S}_- = 0, \mathcal{Z} = 0, D_k = 1|\mathcal{R})\gamma(\mathcal{Z}, D_k) \tag{58}$$

where

$$\gamma(\mathcal{Z}, D_k) = \sum_{S\in\mathcal{Z}} \left(1 - \frac{1}{\lambda_{D_k,S}}\right) \tag{59}$$

where $\mathcal{S}_\pm$ is the set of factual positive (negative) evidenced symptom nodes and $\mathcal{R}$ is the risk factor evidence.

Proof. From the above discussion, the non-zero contributions to the expected disablement are

$$\mathbb{E}(D_k, \mathcal{E})_{\text{dis}} = \sum_{\mathcal{C}\subseteq\mathcal{S}_+} |\mathcal{C}| P(\mathcal{S}_-^* = 0, \mathcal{C}^* = 0, \mathcal{S}_+ \setminus \mathcal{C} = 1 | \mathcal{S}_+, \mathcal{S}_-, \mathcal{R}, \text{do}(D_k^* = 0)) \tag{60}$$

Applying Bayes rule, and noting the the factual evidence states are not children of the intervention node D_k^*, gives

$$\mathbb{E}(D_k, \mathcal{E})_{\text{dis}} = \frac{1}{P(\mathcal{S}_+, \mathcal{S}_- | \mathcal{R})} \sum_{\mathcal{C}\subseteq\mathcal{S}_+} |\mathcal{C}| P(\mathcal{S}_-^* = 0, \mathcal{C}^* = 0, \mathcal{S}_+ \setminus \mathcal{C} = 1, \mathcal{S}_+, \mathcal{S}_- | \mathcal{R}, \text{do}(D_k^* = 0)) \tag{61}$$

Let us now consider the probabilities $Q = P(\mathcal{S}_-^* = 0, \mathcal{C}^* = 0, \mathcal{S} \setminus \mathcal{C}^* = 1, \mathcal{S}_+, \mathcal{S}_- | \mathcal{R}, \text{do}(D_k^* = 0))$. We can express these as marginalizations over the disease layer, which d-separate dual symptom pairs from each-other. First, we express Q in the instance where we assume all $\lambda_{D_k,S} > 0$.

$$\begin{aligned} Q = &\sum_{d,d_k} P(\wedge_{i\neq k} D_i = d_i, D_k = d_k | \mathcal{R}) \prod_{S\in\mathcal{S}_-} P(S^* = 0, S = 0 | \wedge_{i\neq k} D_i = d_i, D_k = d_k, D_k^* = 0) \\ &\times \prod_{S\in\mathcal{C}} P(S^* = 0, S = 1 | \wedge_{i\neq k} D_i = d_i, D_k = d_k, D_k^* = 0) \prod_{S\in\mathcal{S}_+\setminus\mathcal{C}} P(S^* = 1, S = 1 | \wedge_{i\neq k} D_i = d_i, D_k = d_k, D_k^* = 0) \end{aligned} \tag{62}$$

$\mathbb{E}(D_k, \mathcal{E})$ is a sum of products of Q's, therefore if all Q are continuous for $\lambda_{D_k,S} \to 0\ \forall\ S$ we can derive $\mathbb{E}(D_k, \mathcal{E})$ for positive $\lambda_{D_k,S}$ and take the limit $\lambda_{D_k,S} \to 0$ where appropriate. We can consider each term in isolation, as the product of continuous functions is continuous. Each term in Q derives from one of

$$\begin{aligned} &P(s, s^* \mid \wedge_{i\neq k} D_i = d_i, D_k = d_k, \text{do}(D_k^* = 0)) \\ &= \begin{cases} P(s = 0 \mid \wedge_i D_i = d_i) & \text{if } s = s^* = 0 \\ 0 \quad \text{if } s = 0, s^* = 1 \\ \left(\frac{1}{\lambda_{D_k,S}} - 1\right) P(s = 0 \mid \wedge_{i\neq k} D_i = d_i, D_k = 1)\delta(d_k - 1) & \text{if } s = 1, s^* = 0 \text{ and } \lambda_{D_k,S} \neq 0 \\ P(s^{\setminus k} = 0 \mid \wedge_{i\neq k} D_i = d_i, D_k = 1)\delta(d_k - 1) & \text{if } s = 1, s^* = 0 \text{ and } \lambda_{D_k,S} = 0 \\ P(s^{\setminus k} = 1 | \wedge_{i\neq k} D_i = d_i, D_k = 1) & \text{if } s = 1, s^* = 1 \end{cases} \end{aligned} \tag{63}$$

Starting with $P(s = 0 \mid \wedge_i D_i = d_i) = \lambda_{L_S} \prod_{i=1}^N \lambda_{D_i,S}^{d_i}$, this is a linear function of $\lambda_{D_k,S}$ and therefore continuous in the limit $\lambda_{D_k,S} \to 0$. Secondly,

$$\left(\frac{1}{\lambda_{D_k,S}} - 1\right) P(s = 0 \mid \wedge_{i\neq k} D_i = d_i, D_k = 1)\delta(d_k - 1) = \left(\frac{1}{\lambda_{D_k,S}} - 1\right) \lambda_{L_S} \prod_{i=1}^N \lambda_{D_i,S}^{d_i} \delta(d_k - 1) \tag{64}$$

which again is a linear function fo $\lambda_{D_k,S}$ and so is continuous in the limit $\lambda_{D_k,S} \to 0$. $P(s^{\setminus k} = 0 \mid \wedge_{i\neq k} D_i = d_i, D_k = 1)\delta(d_k - 1)$ is a constant function w.r.t $\lambda_{D_k,S}$, as is $P(s^{\setminus k} = 1 | \wedge_{i\neq k} D_i = d_i, D_k = 1)$, so these are also both continuous in the limit.

We therefore proceed under the assumption that $\lambda_{D_k,S} > 0\ \forall\ S$. Applying Lemma 1 simplifies (62) to

$$\begin{aligned} Q = &\sum_{d} P(\wedge_{i\neq k} D_i = d_i, D_k = d_k | \mathcal{R}) \prod_{S\in\mathcal{S}_-} P(S = 0 | \wedge_{i\neq k} D_i = d_i, D_k = d_k) \prod_{S\in\mathcal{C}} P(S = 0 | \wedge_{i\neq k} D_i = d_i, D_k = 1)\delta(d_k - 1) \\ &\times \prod_{S\in\mathcal{S}_+\setminus\mathcal{C}} P(S^{\setminus k} = 1 | \wedge_{i\neq k} D_i = d_i, D_k = 1) \prod_{S\in\mathcal{C}} \left(\frac{1}{\lambda_{D_k,S}} - 1\right) \end{aligned} \tag{65}$$

Note that the only Q that are not multiplied by a factor $|\mathcal{C}| = 0$ in (61) have $\mathcal{C} \neq \emptyset$, and so $\delta(d_k - 1)$ is always present. Marginalizing over all disease states gives

$$Q = P(\mathcal{S}_- = 0, \mathcal{C} = 0, (\mathcal{S}_+ \setminus \mathcal{C})^{\setminus k} = 1, D_k = 1|\mathcal{R}) \prod_{S \in \mathcal{C}} \left(\frac{1}{\lambda_{D_k,S}} - 1 \right) \tag{66}$$

As before, we simplify this using a change of varaibles and the inclusion-exclusion principle. Change variables $\mathcal{C} \to \mathcal{S}_+ \setminus \mathcal{C}$, which along with (66) gives

$$\mathbb{E}(D_k, \mathcal{E})_{\text{dis}} = \frac{1}{P(\mathcal{S}_+, \mathcal{S}_-|\mathcal{R})} \sum_{\mathcal{C} \subseteq \mathcal{S}_+} |\mathcal{S}_+ \setminus \mathcal{C}| P(\mathcal{S}_- = 0, (\mathcal{S}_+ \setminus \mathcal{C}) = 0, \mathcal{C}^{\setminus k} = 1, D_k = 1|\mathcal{R}) \prod_{S \in (\mathcal{S}_+ \setminus \mathcal{C})} \left(\frac{1}{\lambda_{D_k,S}} - 1 \right) \tag{67}$$

Next we apply the inclusion exclusion principle, giving

$$\mathbb{E}(D_k, \mathcal{E})_{\text{dis}} = \frac{1}{P(\mathcal{S}_+, \mathcal{S}_-|\mathcal{R})} \sum_{\mathcal{C} \subseteq \mathcal{S}_+} |\mathcal{S}_+ \setminus \mathcal{C}| \prod_{S \in (\mathcal{S}_+ \setminus \mathcal{C})} \left(\frac{1}{\lambda_{D_k,S}} - 1 \right) \sum_{\mathcal{Z} \subseteq \mathcal{C}} (-1)^{|\mathcal{Z}|} P(\mathcal{S}_- = 0, (\mathcal{S}_+ \setminus \mathcal{C}) = 0, \mathcal{Z}^{\setminus k} = 0, D_k = 1|\mathcal{R}) \tag{68}$$

We can now proceed as before and remove the graph cut operation on the set $\mathcal{Z}$, using the definition of noisy-or (2),

$$\begin{aligned}
P(\mathcal{S}_- = 0, &(\mathcal{S}_+ \setminus \mathcal{C}) = 0, \mathcal{Z}^{\setminus k} = 0, D_k = 1|\mathcal{R}) \\
&= \sum_{d_i, i \neq k} P(\mathcal{S}_- = 0, (\mathcal{S}_+ \setminus \mathcal{C}) = 0, \mathcal{Z}^{\setminus k} = 0, D_k = 1, \wedge_{i \neq k}^N D_i = d_i|\mathcal{R}) \\
&= \sum_{d_i, i \neq k} \prod_{S \in \mathcal{S}_\pm \setminus \mathcal{C}} P(S = 0|D_k = 1, \wedge_{i \neq k}^N D_i = d_i) \prod_{S \in \mathcal{Z}} P(S^{\setminus k} = 0|D_k = 1, \wedge_{i \neq k}^N D_i = d_i) P(D_k = 1, \wedge_{i \neq k}^N D_i = d_i|\mathcal{R}) \\
&= \sum_{d_i, i \neq k} \prod_{S \in \mathcal{S}_\pm \setminus \mathcal{C}} P(S = 0|D_k = 1, \wedge_{i \neq k}^N D_i = d_i) \prod_{S \in \mathcal{Z}} \frac{P(S = 0|D_k = 1, \wedge_{i \neq k}^N D_i = d_i)}{\lambda_{D_k,S}} P(D_k = 1, \wedge_{i \neq k}^N D_i = d_i|\mathcal{R}) \\
&= \frac{P(\mathcal{S}_- = 0, (\mathcal{S}_+ \setminus \mathcal{C}) = 0, \mathcal{Z} = 0, D_k = 1|\mathcal{R})}{\prod_{S \in \mathcal{Z}} \lambda_{D_k,S}}
\end{aligned} \tag{69}$$

Therefore

$$\begin{aligned}
\mathbb{E}(D_k, \mathcal{E})_{\text{dis}} = &\frac{1}{P(\mathcal{S}_+, \mathcal{S}_-|\mathcal{R})} \sum_{\mathcal{C} \subseteq \mathcal{S}_+} |\mathcal{S}_+ \setminus \mathcal{C}| \prod_{S \in \mathcal{S}_+ \setminus \mathcal{C}} \left(\frac{1}{\lambda_{D_k,S}} - 1 \right) \\
&\times \sum_{\mathcal{Z} \subseteq \mathcal{C}} (-1)^{|\mathcal{Z}|} P(\mathcal{S}_- = 0, \mathcal{S}_+ \setminus \mathcal{C} = 0, \mathcal{Z} = 0, D_k = 1|\mathcal{R}) \prod_{S \in \mathcal{Z}} \frac{1}{\lambda_{D_k,S}}
\end{aligned}$$

Finally, we aggregate all terms that have the same symptom marginal. Perform the change of variables $\mathcal{X} = \mathcal{S}_+ \setminus \mathcal{C}$

$$\mathbb{E}(D_k, \mathcal{E})_{\text{dis}} = \frac{1}{P(\mathcal{S}_+, \mathcal{S}_-|\mathcal{R})} \sum_{\mathcal{X} \subseteq \mathcal{S}_+} |\mathcal{X}| \prod_{S \in \mathcal{X}} \left(\frac{1}{\lambda_{D_k,S}} - 1 \right) \sum_{\mathcal{Z} \subseteq \mathcal{S}_+ \setminus \mathcal{X}} (-1)^{|\mathcal{Z}|} P(\mathcal{S}_- = 0, \mathcal{X} = 0, \mathcal{Z} = 0, D_k = 1|\mathcal{R}) \prod_{S \in \mathcal{Z}} \frac{1}{\lambda_{D_k,S}} \tag{70}$$

Clearly each term for a given $\mathcal{X}$ is zero unless $\lambda_{D_k,S} < 1 \; \forall \; S \in \mathcal{X}$, and so we can restrict ourselves to $\mathcal{S} \subseteq \mathcal{S}_+ \cap \mathsf{Ch}(D_k)$. Furthermore, if any $\lambda_{D_k,S} = 0$ for $S \in \mathcal{X}$, then the symptom marginal (which is linearly dependent on $\lambda_{D_k,S}$) is 0 (there is zero probability of observing this symptom to be off if $D_k = 1$), and this term in the sum is zero. Therefore we can restrict the sum to $\mathcal{X} \subseteq S_+^{(k)}(\lambda > 0)$, where $S_+^{(k)}(\lambda > 0)$ is the set of positively evidenced factual symptoms that are children of D_k and have $\lambda_{D_k,S} > 0$. Let $\mathcal{A} = \mathcal{X} \cup \mathcal{Z}$. Each marginal $P(\mathcal{S}_- = 0, \mathcal{A} = 0, D_k = 1|\mathcal{R})$ aggregates a coefficient

$$\frac{1}{P(\mathcal{S}_+, \mathcal{S}_-|\mathcal{R})} \sum_{\mathcal{X} \subseteq \mathcal{A}} |\mathcal{X}| \prod_{S \in \mathcal{X}} \left(\frac{1}{\lambda_{D_k,S}} - 1 \right) (-1)^{|\mathcal{A}| - |\mathcal{X}|} \prod_{S \in \mathcal{A} \setminus \mathcal{X}} \frac{1}{\lambda_{D_k,S}} \tag{71}$$

which simplifies to

$$\frac{1}{P(\mathcal{S}_+,\mathcal{S}_-|\mathcal{R})\prod_{S\in\mathcal{A}}\lambda_{D_k,S}}\sum_{\mathcal{X}\subseteq\mathcal{A}}|\mathcal{X}|(-1)^{|\mathcal{A}|-|\mathcal{X}|}\prod_{S\in\mathcal{X}}(1-\lambda_{D_k,S}) \tag{72}$$

To evaluate this term, define the function

$$G(\mathcal{A}):=\sum_{\mathcal{X}\subseteq\mathcal{A}}|\mathcal{X}|(-1)^{|\mathcal{A}|-|\mathcal{X}|}\prod_{S\in\mathcal{X}}(1-\lambda_{D_k,S}) \tag{73}$$

If we append an element $\{\tilde{S}\}$ to the set $\mathcal{A}$, where $\tilde{S}\notin\mathcal{A}$, we can express $G(\mathcal{A}\cup\{\tilde{S}\})$ as

$$G(\mathcal{A}\cup\{\tilde{S}\})=\sum_{\mathcal{X}\subseteq\mathcal{A}}|\mathcal{X}|(-1)^{|\mathcal{A}|+1-|\mathcal{X}|}\prod_{S\in\mathcal{X}}(1-\lambda_{D_k,S})+\sum_{\mathcal{X}\subseteq\mathcal{A}}(|\mathcal{X}|+1)(-1)^{|\mathcal{A}|+1-|\mathcal{X}|-1}\prod_{S\in\mathcal{X}}(1-\lambda_{D_k,S})\,(1-\lambda_{D_k,\tilde{S}}) \tag{74}$$

where we have split the sum into subsets where containing $\tilde{S}$ and not containing $\tilde{S}$, and then expressed these in terms of the subsets $\mathcal{X}$ of $\mathcal{A}$. This yields the recursive formula

$$G(\mathcal{A}\cup\{\tilde{S}\})=-\lambda_{D_k,\tilde{S}}G(\mathcal{A})+(1-\lambda_{D_k,\tilde{S}})H(\mathcal{A}) \tag{75}$$

where

$$H(\mathcal{A})=\sum_{\mathcal{X}\subseteq\mathcal{A}}(-1)^{|\mathcal{A}|-|\mathcal{X}|}\prod_{S\in\mathcal{X}}(1-\lambda_{D_k,S}) \tag{76}$$

We can determine $H(\mathcal{A})$ by the same technique – noting that

$$\begin{aligned}H(\mathcal{A}\cup\{\tilde{S}\}) &= \sum_{\mathcal{X}\subseteq\mathcal{A}}(-1)^{|\mathcal{A}|+1-|\mathcal{X}|}\prod_{S\in\mathcal{X}}(1-\lambda_{D_k,S})+\sum_{\mathcal{X}\subseteq\mathcal{A}}(-1)^{|\mathcal{A}|+1-|\mathcal{X}|-1}\prod_{S\in\mathcal{X}}(1-\lambda_{D_k,S})\,(1-\lambda_{D_k,\tilde{S}})\\ &= -H(\mathcal{A})+(1-\lambda_{D_k,\tilde{S}})H(\mathcal{A})\\ &= -\lambda_{D_k,\tilde{S}}H(\mathcal{A})\end{aligned}$$

for $\tilde{S}\notin\mathcal{A}$. Then, noting that $H(\emptyset)=1$, we recover

$$H(\mathcal{A})=(-1)^{|\mathcal{A}|}\prod_{S\in\mathcal{A}}\lambda_{D_k,S} \tag{77}$$

and therefore

$$\begin{aligned}G(\mathcal{A}\cup\{\tilde{S}\}) &= -\lambda_{D_k,\tilde{S}}G(\mathcal{A})+(1-\lambda_{D_k,\tilde{S}})(-1)^{|\mathcal{A}|}\prod_{S\in\mathcal{A}}\lambda_{D_k,S}\\ &= (-1)\left[\lambda_{D_k,\tilde{S}}G(\mathcal{A})+(1-\lambda_{D_k,\tilde{S}})(-1)^{|\mathcal{A}\cup\{\tilde{S}\}|}\prod_{S\in\mathcal{A}}\lambda_{D_k,S}\right]\end{aligned}$$

The above recursion relation states that for every new element we append to $\mathcal{A}$, we multiply the previous function by the new $\lambda_{D_k,\tilde{S}}$, add a term with the product of the previous λ's multiplied by $(1-\lambda_{D_k,\tilde{S}})$, and multiply the result by (-1). Starting from $G(\emptyset)=0$ and $G(\{S\})=1-\lambda_{D_k,S}$, it follows that the function must take the form

$$G(\mathcal{A})=(-1)^{|\mathcal{A}|+1}\sum_{S\in\mathcal{A}}(1-\lambda_{D_k,S})\prod_{S'\in\mathcal{A}\backslash S}\lambda_{D_k,S'} \tag{78}$$

Therefore

$$\mathbb{E}(D_k,\mathcal{E})_{\text{dis}} = \frac{1}{P(\mathcal{S}_+,\mathcal{S}_-|\mathcal{R})}\sum_{\mathcal{A}\subseteq\mathcal{S}_+}\frac{1}{\prod\limits_{S\in\mathcal{A}}\lambda_{D_k,S}}(-1)^{|\mathcal{A}|+1}\sum_{S\in\mathcal{A}}(1-\lambda_{D_k,S})\prod_{S'\in\mathcal{A}\setminus S}\lambda_{D_k,S'}\;P(\mathcal{S}_-=0,\mathcal{A}=0,D_k=1|\mathcal{R})$$

$$= \frac{1}{P(\mathcal{S}_+,\mathcal{S}_-|\mathcal{R})}\sum_{\mathcal{A}\subseteq\mathcal{S}_+}(-1)^{|\mathcal{A}|+1}P(\mathcal{S}_-=0,\mathcal{A}=0,D_k=1|\mathcal{R})\sum_{S\in\mathcal{A}}\frac{1-\lambda_{D_k,S}}{\lambda_{D_k,S}} \quad (79)$$

Once again, we have arrived at a corrected form of the standard posterior

$$\mathbb{E}(D_k,\mathcal{E})_{\text{dis}} = \frac{1}{P(\mathcal{S}_+,\mathcal{S}_-|\mathcal{R})}\sum_{\mathcal{A}\subseteq\mathcal{S}_+}(-1)^{|\mathcal{A}|}P(\mathcal{S}_-=0,\mathcal{A}=0,D_k=1|\mathcal{R})\gamma(\mathcal{A},D_k) \quad (80)$$

where

$$\gamma(\mathcal{A},D_k) = |\mathcal{A}| - \sum_{S\in\mathcal{A}}\frac{1}{\lambda_{D_k,S}} \quad (81)$$

and we recover $\mathbb{E}(D_k,\mathcal{E})_{\text{dis}} = P(D_k=1|\mathcal{E})$ in the limit $\gamma(\mathcal{A},D_k)\to 1$.

Finally, consider that for some $S\in\mathcal{A}$, $\lambda_{D_k,S}=0$. Note that $P(\mathcal{S}_-=0,\mathcal{A}=0,D_k=1|\mathcal{R}) = P(\mathcal{S}_-=0,\mathcal{A}=0|\mathcal{R},D_k=1)P(D_k=1|\mathcal{R})$. If any $\lambda_{D_k,S}=0$ for $S\in\mathcal{S}_-$, then this term is 0 by construction.

□

Supplementary note 7: properties of the expected disablement

In this supplementary note we show that the expected disablement satisfies our criteria for diagnostic measures. Although in noisy-or networks the expected disablement coincides with the expected sufficiency, which we have already shown to obey our postulates, we show here that the expected disablement in obeys our postulates in general models - regardless of the choice of graph topology or generative functions.

Theorem 5 (Diagnostic properties of expected disablement). *The expected disablement, defined as*

$$\mathbb{E}(D_k,\mathcal{E})_{dis} := \sum_{\mathcal{S}'}\left|\mathcal{S}_+\setminus\mathcal{S}'_+\right|P(\mathcal{S}'|\mathcal{E},do(D_k=0))$$

satisfies the following three conditions

1. consistency. $\mathbb{E}_{dis}(D_k,\mathcal{E})\propto P(D_k=1|\mathcal{E})$

2. causality. *If* $\nexists\, S\in\mathsf{Dec}(D_k)\cap\mathcal{S}_+\implies\mathbb{E}_{dis}(D_k,\mathcal{E})=0$

3. simplicity. $|\mathbb{E}_{dis}(D_k,\mathcal{E})|\leq|\mathcal{S}_+\cap\mathsf{Dec}(D_k)|$

Proof. First we prove consistency. In the following, we use the notation $*$ to denote counterfactual variables. The term $P(\mathcal{S}'^*|\mathcal{E},\text{do}(D_k^*=0))$ can be expressed as

$$P(\mathcal{S}'^*|\mathcal{E},\text{do}(D_k^*=0)) = \sum_{d_k\in\{0,1\}}P(\mathcal{S}'^*,D_k=d_k|\mathcal{E},\text{do}(D_k^*=0)) \quad (82)$$

$$= \sum_{d_k\in\{0,1\}}P(\mathcal{S}'^*|D_k=d_k,\mathcal{E},\text{do}(D_k^*=0))P(D_k=d_k|\mathcal{E},\text{do}(D_k^*=0)) \quad (83)$$

As D_k is not a descendent of D_k^*, this simplifies to

$$P(\mathcal{S}'^*|\mathcal{E},\text{do}(D_k^*=0)) = \sum_{d_k\in\{0,1\}}P(\mathcal{S}'^*|D_k=d_k,\mathcal{E},\text{do}(D_k^*=0))P(D_k=d_k|\mathcal{E}) \quad (84)$$

If $D_k=0$ then the factual and counterfactual symptoms have identical states on their parents, and therefor are copies of each other. As a result, $\mathcal{S}_+=\mathcal{S}'_+$ and the expected disablement is identical to 0. The only term that is non-zero is therefore when $D_k=1$, and all non-zero terms in (84) therefore have a coefficient of

$P(D_k = 1|\mathcal{E})$. To see that causality is satisfied, note that $\left|\mathcal{S}_+ \setminus \mathcal{S}'_+\right| \neq 0$ iff $\mathcal{S}'_+ \subset \mathcal{S}_+$, which requires that at least one symptom has been switched off. If D_k is not a parent of any $\mathcal{S}_+$, then $P(\mathcal{S}'^*|\mathcal{E}, \text{do}(D^*_k = 0)) = 0$ unless $\mathcal{S}'^* = \mathcal{S}$ (the symptom evidence is unchanged), which implies that $\left|\mathcal{S}_+ \setminus \mathcal{S}'_+\right| = 0$, satisfying causality. Finally, note that $\mathbb{E}_{\text{dis}}(D_k, \mathcal{E})$ is a convex combination over the values of the set difference function $\left|\mathcal{S}_+ \setminus \mathcal{S}'_+\right|$, and therefore is upper bounded by $\mathbb{E}_{\text{dis}}(D_k, \mathcal{E}) \leq |\mathcal{S}_+|$, the number of positively evidenced symptoms that are children of D_k. Therefore, the expected disablement is upper bounded by the maximal number of positive symptoms that can be caused by D_k.

□

Supplementary note 8: relation to other counterfactual measures

In this supplementary note we compare the expected disablement and expected sufficiency to three related counterfactual measures; the effect of treatment on the treated [3], the probability of sufficiency and the probability of necessity [16]. We briefly discuss these measures and their applicability to the task of diagnosis.

We are interested in quantifying the causal relations between diseases and symptoms—the degree to which a (latent) disease causes the (observed) symptoms. In this context, the effect of treatment on the treated (ETT), probability of necessity (PN) and probability of sufficiency (PS) for a disease-symptom pair (D, S) are defined as,

$$\text{ETT} = P(S^* = 1|D = 1, \text{do}(D^* = 0)) \tag{85}$$
$$\text{PN} = P(S^* = 0|S = 1, D = 1, \text{do}(D^* = 0)) \tag{86}$$
$$\text{PS} = P(S^* = 1|S = 0, D = 0, \text{do}(D^* = 1)) \tag{87}$$

The ETT measures the probability that symptom S would be present, given that the disease is present, had the disease not been present. Unlike the expected disablement and sufficiency, this counterfactual measure can be identified from data under causal assumptions, without requiring knowledge of the underlying structural equations [25]. Note that this query contains no information as to the factual state of the patients symptoms (whether or not the patient presented symptom S). In other words, the ETT measures the causal effect of D on S 'on average', and provides the same value regardless of whether or not S is present. Incorporating knowledge of the patients symptoms is vital for diagnosis, and as the ETT is not capable of incorporating symptom evidence it is not suitable for diagnosis.

The PN quantifies the likelihood that observed event $D = 1$ caused $S = 1$. However, it includes the observation that $D = 1$, whereas in diagnostic tasks the disease is (typically) unobserved. Furthermore, it does not incorporate evidence for symptoms that are not present ($S = 0$). Absent symptoms are important diagnostic determinants and should not be disregarded by a desirable diagnostic measure. Likewise, the PS includes the observation that $D = 0$, and cannot incorporate positive symptom evidence.

Relating the expected disablement (sufficiency) to these measures, it can best be understood as a generalization of the probability of necessity (probability of sufficiency) such that i) it incorporates posterior inference on the disease state, rather than treating it as observed, and ii) it computes an expectation over symptoms states, such that it favours diseases the explain many symptoms, and iii) it incorporates both positive and negative symptom evidence (as well as evidence on variables that do not correspond to the cause D or the effect S, such as risk factors).

Supplementary note 9: examples of diagnosing with the posterior, expected disablement and sufficiency

In this supplementary note we look at the expected disablement, sufficiency and the posterior for some simple toy models. This serves to highlight cases where using associative inference (the posterior) to diagnose diseases results in different diagnoses than if we were to use the expected disablement and expected sufficiency. It also provides some indication as to why the expected disablement and sufficiency achieve a similar accuracy on our test set.

First, we look at a simple PGM where the posterior can return a spurious diagnoses. Consider the following toy model,

There is a symptom S that is generated by a set of diseases $D_1, \ldots, D_N$, and these diseases share a common risk factor R. R is also a cause of another disease D_X which does not cause S. First, consider the evidence set $\mathcal{E} = \{S = 1\}$. Diseases are typically rare, $P(D_i = 1) \ll 1$, and so it is likely that the patient has one of the diseases $D_1, D_2, \ldots, D_N$ (as one is needed to explain $S = 1$), but very unlikely that the patient has multiple diseases (as only one disease is required to explain $S = 1$. This causes diseases $D_1, D_2, \ldots, D_N$ to compete to explain $S = 1$, diluting of the diseases posteriors $P(D_i|E)$ for $i = 1, 2, \ldots, N$ in a phenomena know as 'explaining away' [26]. As a result, the single disease posteriors can be quite small as the number of competing diseases N increases, $P(D_i = 1||\mathcal{E}) \sim 1/N$. As a consequence of $p(\text{any}\{D_i = 1\}_{i=1}^N|\mathcal{E}) \approx 1$, the risk factor posterior can be large, $p(R = 1|\mathcal{E}) \approx 1$, as R is capable of explaining all $D_1, \ldots, D_N$. If disease D_X has a strong enough association with R, then it can end up with the largest posterior, $p(D_X = 1|\mathcal{E}) > p(D_i = 1|\mathcal{E})$ for all $i = 1, \ldots, N$, despite it being impossible that D_X is causing the observed symptoms.

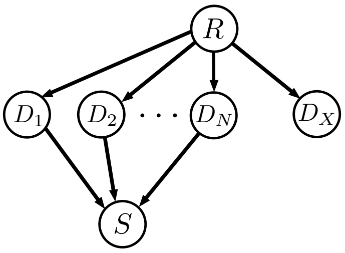

Supplementary Figure 5: Example of diagnostic model with back-door paths

This is an example of *confounding* [27], where the presence of a latent risk factor R generates correlations between D_X and S. Note that, even if R was observed, D_X can have the largest posterior out of all the diseases, if its association with R is strong enough, i.e. $P(D_X = 1|S = 1, R = 1) = P(D_X = 1|R = 1)$ (due to d-separation of D_X from S given R) can be large if $P(D_X = 1|R = 1) \approx 1$. In this case, the posterior still returns a spurious diagnosis despite there being no confounding. These examples are similar to Example 1 in the section **Associative diagnosis**.

Next, we consider a class of Bayesian models that allow us to compute simple expressions for the posterior, expected disablement and expected sufficiency. These are two layer noisy-OR diagnostic networks (also know as BN20 networks [28]), which have a single layer of independent diseases $D_1, \ldots, D_N$ and symptoms generated from these diseases by noisy-OR CPTs. An depiction of these networks is shown below,

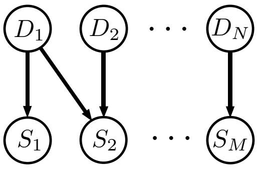

Supplementary Figure 6: Two layer noisy-OR diagnostic network

Consider the case where the evidence is a single positive symptoms $\mathcal{E} = \{S = 1\}$. The posterior of a single disease $P(D_i = 1|S = 1)$ can be calculated as,

$$P(D_k = 1|S = 1) = \frac{P(S = 1, D_k = 1)}{P(S = 1)} \tag{88}$$

$$= \frac{(1 - P(S = 0|D_k = 1))\, P(D_k = 1)}{P(S = 1)} \tag{89}$$

$$= \frac{P(D_k = 1)}{P(S = 1)} \left(1 - \frac{P(S = 0)\lambda_{k,s}}{P(D_k = 1)\lambda_{k,s} + P(D_k = 0)}\right) \tag{90}$$

where in (90) we have used the Quickscore formulas [10],

$$P(S = 0) = \prod_{i=1}^{N} \left[P(D_i = 1)\lambda_{i,s} + P(D_i = 0)\right] \tag{91}$$

$$P(S = 0, D_k = 1) = \prod_{\substack{i=1 \\ i \neq k}}^{N} \left[P(D_i = 1)\lambda_{i,s} + P(D_i = 0)\right] P(D_k = 1)\lambda_{k,s} \tag{92}$$

The expected disablement and sufficiency given by Theorem 2,

$$\mathbb{E}_{\text{dis}} = \frac{P(D_k = 1) \times 0 - P(S = 0, D_k = 1) \times \left(1 - \frac{1}{\lambda_{k,s}}\right)}{P(S = 1)} \tag{93}$$

$$= \left(\frac{1}{\lambda_{k,s}} - 1\right) \frac{P(S = 0, D_k = 1)}{P(S = 1)} \tag{94}$$

$$\mathbb{E}_{\text{suff}} = \frac{P(D_k = 1) \times (1 - \lambda_{k,s}) - P(S = 0, D_k = 1) \times 0}{P(S = 1)} \tag{95}$$

$$= \frac{P(D_k = 1)(1 - \lambda_{k,s})}{P(S = 1)} \tag{96}$$

The expected sufficiency ranks diseases as,

$$\mathbb{E}_{\text{suff}}(D_i, \mathcal{E}) \geq \mathbb{E}_{\text{suff}}(D_j, \mathcal{E}) \tag{97}$$

$$\therefore \frac{P(D_i = 1)(1 - \lambda_{i,s})}{P(S = 1)} \geq \frac{P(D_j = 1) \times (1 - \lambda_{j,s})}{P(S = 1)} \tag{98}$$

$$\therefore P(D_i = 1)(1 - \lambda_{i,s}) \geq P(D_j = 1)(1 - \lambda_{j,s}) \tag{99}$$

The expected disablement ranks diseases as,

$$\mathbb{E}_{\text{dis}}(D_i, \mathcal{E}) \geq \mathbb{E}_{\text{dis}}(D_j, \mathcal{E}) \tag{100}$$

$$\therefore \left(\frac{1}{\lambda_{i,s}} - 1\right) \frac{P(S = 0, D_i = 1)}{P(S = 1)} \geq \left(\frac{1}{\lambda_{j,s}} - 1\right) \frac{P(S = 0, D_j = 1)}{P(S = 1)} \tag{101}$$

$$\therefore \left(\frac{1}{\lambda_{i,s}} - 1\right) \frac{P(S = 0)P(D_i = 1)\lambda_{i,s}}{(P(D_i = 1)\lambda_{i,s} + P(D_i = 0))} \geq \left(\frac{1}{\lambda_{j,s}} - 1\right) \frac{P(S = 0)P(D_j = 1)\lambda_{j,s}}{(P(D_j = 1)\lambda_{j,s} + P(D_j = 0))} \tag{102}$$

where in (102) we have applied equations (91) and (92). It is simple to show that this inequality simplifies to,

$$P(D_i = 1)(1 - \lambda_{i,s}) \geq P(D_j = 1)(1 - \lambda_{j,s}) \tag{103}$$

Therefore in this example, for two layer noisy-OR models, the expected disablement and expected sufficiency return the same disease rankings (though their values for a given disease will differ). These two layer networks are identical to our three layer networks in the limit that there are no correlations between diseases. This suggests why we observe very similar disease rankings (and hence accuracy) for the expected disablement and sufficiency in our experiments; because symptoms in our model follow noisy-OR statistics and diseases in our model are only weakly correlated by latent risk factors.

Lets now compare these rankings to the posterior ranking in our example of a two layer network with $\mathcal{E} = \{S = T\}$, i.e.

$$P(D_i = 1|\mathcal{E}) \geq P(D_j = 1|\mathcal{E}) \tag{104}$$

$$\implies P(D_i = 1)\left(1 - \frac{P(S = 0)\lambda_{i,s}}{P(D_i = 1)\lambda_{i,s} - P(D_i = 0)}\right) \geq P(D_j = 1)\left(1 - \frac{P(S = 0)\lambda_{j,s}}{P(D_j = 1)\lambda_{j,s} - P(D_j = 0)}\right) \tag{105}$$

compared to,

$$\mathbb{E}_{\text{dis/suff}}(D_i, \mathcal{E}) \geq \mathbb{E}_{\text{dis/suff}}(D_j, \mathcal{E}) \tag{106}$$

$$\implies P(D_i = 1)(1 - \lambda_{i,s}) \geq P(D_j = 1)(1 - \lambda_{j,s}) \tag{107}$$

First, note that if $\lambda_{i,s} = 1$, which is equivalent to D_i not being a cause of S, then $\mathbb{E}_{\text{dis}} = \mathbb{E}_{\text{suff}} = 0$ and this disease can never be ranked higher than any disease where $\lambda_{j,s} < 1$. This is not the case for the posterior, where (105) reduces to

$$P(D_i = 1)P(S = 1) \geq P(D_j = 1)\left(1 - \frac{P(S = 0)\lambda_{j,s}}{P(D_j = 1)\lambda_{j,s} - P(D_j = 0)}\right) \tag{108}$$

I.e. the posterior can favour D_i over D_j simply because it has a larger prior, even if it cannot be a cause of $S = T$. Therefore, even in 2 layer networks where there are no confounders between diseases, the posterior can return a spurious diagnosis. Note that simply ignoring diseases that are not direct causes of any of the patients symptoms is insufficient. In general a disease D_i may have some causal relation to the symptoms $\lambda_{i,s} \neq 1$, but this could be a weak relation, $\lambda_{i,s} = 1 - \epsilon$ where $\epsilon \ll 1$. Such a disease can still have a large posterior—for example, a disease with a high prevalence but which only weakly causes the patients symptoms.

Supplementary note 10: example clinical vignette

This supplementary note presents an example of the clinical vignettes used in our experiments. Details of the vignettes authorship have been removed. Details on the evidence that has been included comprises of the medical concept, whether or not it is included in our disease model, and whether or not it is present in the patient.


```
{
  age: 60,
  diseases: Carcinoid tumour
  duration: Months,
  gender: Male,
  initial_input: I am suffering from facial flushing ,
  risk_factors: [
    {name: Dyslipidemia
    in model: True
    presence: present},
    {name: Essential hypertension
    in model: True
     presence: present},
    {name: Ex−smoker
    in model: True
     presence: present},
    {name: Family history of diabetes mellitus type 2
    in model: True
     presence: present},
    {name: Family history of bowel cancer
    in model: True
     presence: present}
    ],
  symptoms: [
    {name: Flushing
    in model: True
    presence: present},
    {name: Diarrhea
    in model: True
    presence: present},
    {name: Facial redness
    in model: False
    presence: present},
    {name: Flushing worse with exercise and stress
    in model: False
    presence: present},
    {name: Flushing triggered by alcohol, chocolate and bananas
    in model: False
    presence: present},
    {name: Fresh blood PR (hematochezia)
    in model: True
    presence: present},
    {name: Cramping Generalized Abdominal Pain
    in model: True
    presence: present},
    {name: Palpitations
    in model: True
    presence: not present},
    {name: Unintentional weight loss
    in model: True
    presence: not present},
    {name: Wheezing
    in model: True
    presence: not present}
  ],
}
```


supplementary tables

In this supplementary note we list the results of experiments 1 and 2. Experiment 1 compares the top k accuracy of our algorithms. In experiment 2 we compare the diagnostic accuracy of 44 doctors to our associative (Bayesian) and counterfactual diagnostic algorithms. The table below records the scores of each doctor and the associative and counterfactual algorithm shadowing them.

TABLE I: Results for experiment 1: table shows the top k accuracy for the posterior, expected disablement and expected sufficiency ranking algorithms, for N from 1 to 15.

N	Posterior	Disablement	Sufficiency
1	0.509 ± 0.012	0.536 ± 0.012	0.534 ± 0.012
2	0.652 ± 0.012	0.702 ± 0.011	0.703 ± 0.011
3	0.735 ± 0.011	0.784 ± 0.01	0.785 ± 0.01
4	0.785 ± 0.01	0.829 ± 0.009	0.829 ± 0.009
5	0.823 ± 0.009	0.867 ± 0.008	0.87 ± 0.008
6	0.849 ± 0.009	0.894 ± 0.008	0.894 ± 0.008
7	0.868 ± 0.008	0.91 ± 0.007	0.91 ± 0.007
8	0.882 ± 0.008	0.917 ± 0.007	0.914 ± 0.007
9	0.893 ± 0.008	0.925 ± 0.006	0.924 ± 0.006
10	0.899 ± 0.007	0.93 ± 0.006	0.929 ± 0.006
11	0.908 ± 0.007	0.936 ± 0.006	0.937 ± 0.006
12	0.916 ± 0.007	0.944 ± 0.006	0.943 ± 0.006
13	0.923 ± 0.007	0.948 ± 0.005	0.947 ± 0.005
14	0.926 ± 0.006	0.951 ± 0.005	0.95 ± 0.005
15	0.928 ± 0.006	0.954 ± 0.005	0.954 ± 0.005
16	0.932 ± 0.006	0.957 ± 0.005	0.958 ± 0.005
17	0.935 ± 0.006	0.961 ± 0.005	0.962 ± 0.005
18	0.937 ± 0.006	0.963 ± 0.005	0.963 ± 0.005
19	0.941 ± 0.006	0.967 ± 0.004	0.967 ± 0.004
20	0.944 ± 0.006	0.968 ± 0.004	0.968 ± 0.004

TABLE II: Results for experiment 1: table shows the mean position of the true disease for the associative (A) and counterfactual (C, expected sufficiency) algorithms over all 1671 cases. Results are stratified over the rareness of the disease (given the age and gender of the patient). For each disease rareness category, the number of cases N is given. Also the number of cases where the associative algorithm ranked the true disease higher than the counterfactual algorithm (Wins (A)), the counterfactual algorithm ranked the true disease higher than the associative algorithm (Wins (C)), and the number of cases where the two algorithms ranked the true disease in the same position (Draws) are given, for all cases and for each disease rareness class.

	**Vignettes**						
	**All**	**Very common**	**Common**	**Uncommon**	**Rare**	**Very rare**	**Extremely rare**
N	1671	131	413	546	353	210	18
Mean position (A)	3.81 ± 5.25	2.85 ± 4.27	2.71 ± 3.86	3.72 ± 5.05	4.35 ± 5.28	5.45 ± 6.52	4.22 ± 5.19
Mean position (C)	3.16 ± 4.40	2.5 ± 3.55	2.32 ± 3.25	3.01 ± 4.07	3.72 ± 4.74	4.38 ± 5.53	3.56 ± 3.96
Wins (A)	31	2	7	9	9	4	0
Wins (C)	412	20	80	135	103	69	5
Draws	1228	109	326	402	241	137	13

TABLE III: Results for experiment 2: table shows the accuracy obtained by the doctor and each algorithm shadowing the doctors, for each of the 44 single-doctor experiments. The accuracies are reported with the standard standard deviation of the mean estimator.

Doctor number	Doctor accuracy	Posterior	Expected sufficiency	Expected disablement
0	0.725 ± 0.019	0.656 ± 0.02	0.694 ± 0.019	0.692 ± 0.019
1	0.823 ± 0.022	0.719 ± 0.026	0.771 ± 0.025	0.774 ± 0.025
2	0.89 ± 0.018	0.791 ± 0.023	0.834 ± 0.021	0.837 ± 0.021
3	0.805 ± 0.023	0.811 ± 0.023	0.834 ± 0.021	0.831 ± 0.022
4	0.776 ± 0.034	0.855 ± 0.029	0.908 ± 0.023	0.914 ± 0.023
5	0.612 ± 0.028	0.779 ± 0.024	0.827 ± 0.022	0.834 ± 0.021
6	0.799 ± 0.02	0.739 ± 0.022	0.794 ± 0.02	0.794 ± 0.02
7	0.778 ± 0.026	0.767 ± 0.026	0.825 ± 0.024	0.825 ± 0.024
8	0.69 ± 0.025	0.788 ± 0.022	0.833 ± 0.02	0.833 ± 0.02
9	0.698 ± 0.058	0.81 ± 0.049	0.873 ± 0.042	0.873 ± 0.042
10	0.905 ± 0.037	0.841 ± 0.046	0.873 ± 0.042	0.889 ± 0.04
11	0.783 ± 0.034	0.72 ± 0.038	0.797 ± 0.034	0.797 ± 0.034
12	0.684 ± 0.053	0.75 ± 0.05	0.789 ± 0.047	0.776 ± 0.048
13	0.627 ± 0.063	0.712 ± 0.059	0.78 ± 0.054	0.78 ± 0.054
14	0.788 ± 0.033	0.737 ± 0.035	0.776 ± 0.033	0.782 ± 0.033
15	0.891 ± 0.018	0.73 ± 0.025	0.776 ± 0.024	0.776 ± 0.024
16	0.791 ± 0.043	0.835 ± 0.039	0.879 ± 0.034	0.879 ± 0.034
17	0.651 ± 0.051	0.767 ± 0.046	0.802 ± 0.043	0.802 ± 0.043
18	0.722 ± 0.043	0.806 ± 0.038	0.833 ± 0.036	0.833 ± 0.036
19	0.75 ± 0.056	0.717 ± 0.058	0.767 ± 0.055	0.783 ± 0.053
20	0.566 ± 0.068	0.642 ± 0.066	0.66 ± 0.065	0.66 ± 0.065
21	0.797 ± 0.026	0.73 ± 0.029	0.776 ± 0.027	0.781 ± 0.027
22	0.671 ± 0.03	0.667 ± 0.03	0.736 ± 0.028	0.735 ± 0.028
23	0.695 ± 0.032	0.67 ± 0.033	0.709 ± 0.032	0.708 ± 0.032
24	0.735 ± 0.035	0.71 ± 0.036	0.781 ± 0.033	0.774 ± 0.034
25	0.648 ± 0.047	0.705 ± 0.045	0.752 ± 0.042	0.752 ± 0.042
26	0.7 ± 0.065	0.66 ± 0.067	0.66 ± 0.067	0.66 ± 0.067
27	0.854 ± 0.035	0.777 ± 0.041	0.835 ± 0.037	0.835 ± 0.037
28	0.787 ± 0.039	0.778 ± 0.04	0.824 ± 0.037	0.815 ± 0.037
29	0.636 ± 0.048	0.697 ± 0.046	0.747 ± 0.044	0.747 ± 0.044
30	0.604 ± 0.046	0.739 ± 0.042	0.748 ± 0.041	0.748 ± 0.041
31	0.758 ± 0.053	0.818 ± 0.047	0.909 ± 0.035	0.908 ± 0.036
32	0.825 ± 0.039	0.691 ± 0.047	0.711 ± 0.046	0.701 ± 0.046
33	0.5 ± 0.065	0.633 ± 0.062	0.683 ± 0.06	0.683 ± 0.06
34	0.607 ± 0.063	0.607 ± 0.063	0.689 ± 0.059	0.689 ± 0.059
35	0.574 ± 0.063	0.623 ± 0.062	0.689 ± 0.059	0.689 ± 0.059
36	0.55 ± 0.064	0.633 ± 0.062	0.667 ± 0.061	0.667 ± 0.061
37	0.61 ± 0.063	0.576 ± 0.064	0.661 ± 0.062	0.661 ± 0.062
38	0.592 ± 0.04	0.697 ± 0.037	0.724 ± 0.036	0.715 ± 0.037
39	0.708 ± 0.044	0.67 ± 0.046	0.717 ± 0.044	0.708 ± 0.044
40	0.702 ± 0.045	0.721 ± 0.044	0.74 ± 0.043	0.74 ± 0.043
41	0.765 ± 0.059	0.765 ± 0.059	0.824 ± 0.053	0.824 ± 0.053
42	0.639 ± 0.053	0.723 ± 0.049	0.783 ± 0.045	0.768 ± 0.047
43	0.704 ± 0.054	0.648 ± 0.057	0.704 ± 0.054	0.704 ± 0.054

Supplementary references

[1] A. Balke and J. Pearl, "Counterfactual probabilities: Computational methods, bounds and applications," in *Proceedings of the Tenth international conference on Uncertainty in artificial intelligence*, pp. 46–54, Morgan Kaufmann

Publishers Inc., 1994.

[2] S. L. Lauritzen, *Graphical models*, vol. 17. Clarendon Press, 1996.

[3] J. Pearl, *Causality*. Cambridge university press, 2009.

[4] P. Spirtes and K. Zhang, "Causal discovery and inference: concepts and recent methodological advances," in *Applied informatics*, vol. 3, p. 3, Springer, 2016.

[5] C. M. Lee, C. Hart, J. G. Richens, and S. Johri, "Leveraging directed causal discovery to detect latent common causes," *preprint at https://arxiv.org/abs/1910.10174*, 2019.

[6] M. A. Shwe, B. Middleton, D. E. Heckerman, M. Henrion, E. J. Horvitz, H. P. Lehmann, and G. F. Cooper, "Probabilistic diagnosis using a reformulation of the internist-1/qmr knowledge base," *Methods of information in Medicine*, vol. 30, no. 04, pp. 241–255, 1991.

[7] R. Miller, "A history of the internist-1 and quick medical reference (qmr) computer-assisted diagnosis projects, with lessons learned," *Yearbook of medical informatics*, vol. 19, no. 01, pp. 121–136, 2010.

[8] Z. Yongli, H. Limin, and L. Jinling, "Bayesian networks-based approach for power systems fault diagnosis," *IEEE Transactions on Power Delivery*, vol. 21, no. 2, pp. 634–639, 2006.

[9] D. Nikovski, "Constructing bayesian networks for medical diagnosis from incomplete and partially correct statistics," *IEEE Transactions on Knowledge & Data Engineering*, no. 4, pp. 509–516, 2000.

[10] D. Heckerman, "A tractable inference algorithm for diagnosing multiple diseases," in *Machine Intelligence and Pattern Recognition*, vol. 10, pp. 163–171, Elsevier, 1990.

[11] Y. Liu, K. Liu, and M. Li, "Passive diagnosis for wireless sensor networks," *IEEE/ACM Transactions on Networking (TON)*, vol. 18, no. 4, pp. 1132–1144, 2010.

[12] Y. Halpern and D. Sontag, "Unsupervised learning of noisy-or bayesian networks," *preprint at https://arxiv.org/abs/1309.6834*, 2013.

[13] L. Perreault, S. Strasser, M. Thornton, and J. W. Sheppard, "A noisy-or model for continuous time bayesian networks.," in *FLAIRS Conference*, pp. 668–673, 2016.

[14] S. Arora, R. Ge, T. Ma, and A. Risteski, "Provable learning of noisy-or networks," in *Proceedings of the 49th Annual ACM SIGACT Symposium on Theory of Computing*, pp. 1057–1066, ACM, 2017.

[15] A. Abdollahi and K. Pattipati, "Unification of leaky noisy or and logistic regression models and maximum a posteriori inference for multiple fault diagnosis using the unified model," in *DX Conference (Denver, Co)*, 2016.

[16] J. Pearl, "Probabilities of causation: three counterfactual interpretations and their identification," *Synthese*, vol. 121, no. 1-2, pp. 93–149, 1999.

[17] I. Shpitser and J. Pearl, "What counterfactuals can be tested," in *Proceedings of the Twenty-Third Conference on Uncertainty in Artificial Intelligence*, pp. 352–359, AUAI Press, 2007.

[18] Y. Perov, L. Graham, K. Gourgoulias, J. G. Richens, C. M. Lee, A. Baker, and S. Johri, "Multiverse: Causal reasoning using importance sampling in probabilistic programming," *preprint at https://arxiv.org/abs/1910.08091*, 2019.

[19] J. Y. Halpern, *Actual causality*. MiT Press, 2016.

[20] J. Y. Halpern and J. Pearl, "Causes and explanations: A structural-model approach. part ii: Explanations," *The British journal for the philosophy of science*, vol. 56, no. 4, pp. 889–911, 2005.

[21] J. Y. Halpern, "Axiomatizing causal reasoning," *Journal of Artificial Intelligence Research*, vol. 12, pp. 317–337, 2000.

[22] T. Eiter and T. Lukasiewicz, "Complexity results for structure-based causality," *Artificial Intelligence*, vol. 142, no. 1, pp. 53–89, 2002.

[23] J. Pearl *et al.*, "Causal inference in statistics: An overview," *Statistics surveys*, vol. 3, pp. 96–146, 2009.

[24] I. Shpitser and J. Pearl, "Effects of treatment on the treated: Identification and generalization," in *Proceedings of the twenty-fifth conference on uncertainty in artificial intelligence*, pp. 514–521, AUAI Press, 2009.

[25] I. Shpitser and J. Pearl, "Effects of treatment on the treated: Identification and generalization," *preprint at https://arxiv.org/abs/1205.2615*, 2012.

[26] M. P. Wellman and M. Henrion, "Explaining'explaining away'," *IEEE Transactions on Pattern Analysis and Machine Intelligence*, vol. 15, no. 3, pp. 287–292, 1993.

[27] J. Pearl, "Comment understanding simpson's paradox," *The American Statistician*, vol. 68, no. 1, pp. 8–13, 2014.

[28] Q. Morris, "Recognition networks for approximate inference in bn20 networks," in *Proceedings of the Seventeenth conference on Uncertainty in artificial intelligence*, pp. 370–377, Morgan Kaufmann Publishers Inc., 2001.
