# Supplementary material for: Improving the accuracy of medical diagnosis with causal machine learning
Source: Nat Commun. 2020 Aug 11;11:3923. doi: 10.1038/s41467-020-17419-7 (PMC7419549; doi:10.1038/s41467-020-17419-7)
Supplement: Supplementary file 4 — Source Data [file 41467_2020_17419_MOESM4_ESM.zip › Code submission/README.rtf]

InformationCode for generating all graphs, tables and experimental results included in manuscriptThis document is for the purposes of manuscript submission and the nature communications editorial staff and should not be viewed and distributed outside of the nature communications editorial staffThe code is not optimised to be run on a ``normal desktop computer’’ and should be run on a CPU clusterRequirementsrequests==2.23.0pandas==0.24.2numpy==1.18.2matplotlib==3.2.1
